# Cyanovirin-N binds to select SARS-CoV-2 spike oligosaccharides outside of the receptor binding domain and blocks infection by SARS-CoV-2

**DOI:** 10.1073/pnas.2214561120

**Published:** 2023-02-28

**Authors:** Jordana Muñoz-Basagoiti, Fábio Luís Lima Monteiro, Lauren R. H. Krumpe, Victoria Armario-Najera, Shilpa R. Shenoy, Daniel Perez-Zsolt, Harrison James Westgarth, Gemma Villorbina, Larissa Maciel Bomfim, Dàlia Raïch-Regué, Lara Nogueras, Curtis J. Henrich, Marçal Gallemí, Filipe Romero Rebello Moreira, Pascual Torres, Jennifer Wilson, Mirela D’arc, Silvia Marfil, Alice Laschuk Herlinger, Edwards Pradenas, Luiza Mendonça Higa, Manuel Portero-Otin, Benjamin Trinité, Richard M. Twyman, Teresa Capell, Amilcar Tanuri, Julià Blanco, Nuria Izquierdo-Useros, Elibio L. Rech, Paul Christou, Barry R. O’Keefe

**Affiliations:** ^a^IrsiCaixa Acquired Immune Deficiency Syndrome Research Institute, Badalona 08916, Spain; ^b^Laboratory of Molecular Virology, Institute of Biology, Department of Genetics, Federal University of Rio de Janeiro, Rio de Janeiro 21941-90, Brazil; ^c^Molecular Targets Program, Center for Cancer Research, National Cancer Institute-Frederick, NIH, Frederick, MD 21702; ^d^Basic Science Program, Leidos Biomedical Research, Inc., Frederick National Laboratory for Cancer Research, Frederick, MD 21702; ^e^Department of Crop and Forest Sciences, University of Lleida-Agrotecnio Center, Lleida 25198, Spain; ^f^Laboratory of Diversity and Viral Diseases, Institute of Genetics, Federal University of Rio de Janeiro, Rio de Janeiro 21941-90, Brazil; ^g^Twyman Research Management Ltd, Scarborough YO11 9FJ, UK; ^h^Germans Trias i Pujol Research Institute, Badalona 08916, Spain; ^i^Centro de Investigación Biomédica en Red Enfermedades Infecciosas, Madrid 28029, Spain; ^j^Universitat de Vic - Universitat Central de Catalunya, Vic 08500, Spain; ^k^Embrapa Genetic Resources and Biotechnology National Institute of Science and Technology in Synthetic Biology, Brasília 70770-917, Brazil; ^l^ICREA, Catalan Institute for Research and Advanced Studies, Barcelona 08010, Spain; ^m^Natural Products Branch, Developmental Therapeutics Program, Division of Cancer Treatment and Diagnosis, National Cancer Institute, Frederick, MD 21702

**Keywords:** antiviral, SARS-CoV-2, lectin, spike glycoprotein

## Abstract

Severe acute respiratory syndrome coronavirus 2 (SARS-CoV-2) is an enveloped positive stranded RNA virus which has caused the recent deadly pandemic called COVID-19. The SARS-CoV-2 virion is coated with a heavily glycosylated Spike glycoprotein which is responsible for attachment and entry into target cells. One, as yet unexploited strategy for preventing SARS-CoV-2 infections, is the targeting of the glycans on Spike. Lectins are carbohydrate-binding proteins produced by plants, algae, and cyanobacteria. Some lectins can neutralize enveloped viruses displaying external glycoproteins, offering an alternative therapeutic approach for the prevention of infection with virulent β-coronaviruses, such as SARS-CoV-2. Here we show that the cyanobacterial lectin cyanovirin-N (CV-N) can selectively target SARS-CoV-2 Spike oligosaccharides and inhibit SARS-CoV-2 infection in vitro and in vivo. CV-N neutralizes Delta and Omicron variants in vitro better than earlier circulating viral variants. CV-N binds selectively to Spike with a Kd as low as 15 nM and a stoichiometry of 2 CV-N: 1 Spike but does not bind to the receptor binding domain (RBD). Further mapping of CV-N binding sites on Spike shows that select high-mannose oligosaccharides in the S1 domain of Spike are targeted by CV-N. CV-N also reduced viral loads in the nares and lungs in vivo to protect hamsters against a lethal viral challenge. In summary, we present an anti-coronavirus agent that works by an unexploited mechanism and prevents infection by a broad range of SARS-CoV-2 strains.

## Significance

The antiviral lectin cyanovirin-N (CV-N) is shown to have potent activity against the syndromecoronavirus 2 (SARS-CoV-2) virus. CV-N showed improved binding and potency against more recent SARS-CoV-2 variants of concern and is shown to be able to reduce the severity of SARS-CoV-2 infections in test animals. The mechanism of action for CV-N is distinct from that of currently used anti-COVID therapeutics or from common vaccine targets on the receptor binding domain. CV-N is a potential broad-spectrum agent against infection from SARS-CoV-2.

Severe acute respiratory syndrome coronavirus 2 (SARS-CoV-2) is a novel coronavirus responsible for the human pandemic of coronavirus disease 2019 (COVID-19) that, as of January 6, 2023, has infected more than 650 million people and caused more than 6.6 million deaths (1). Although there is a broad current effort to distribute multiple vaccines based on recombinant viral nucleic acids, proteins, and peptides, these can reduce death risk but not viral transmission, enabling viral circulation and disease incidence (2, 3). In addition, the emergence of SARS-CoV-2 highly transmissible variants that overcome the immunological barrier induced by SARS-CoV-2 vaccines and cause disease in fully vaccinated individuals (4) imposes challenges for the use of immunological agents, such as vaccines and monoclonal neutralizing antibody therapy, in the prophylaxis and prevention of COVID-19. Significant reductions in the efficacy of monoclonal antibody treatments for SARS-CoV-2 infections resulted from the SARS-CoV-2 variant of concern (VOC) Omicron (5). Some of this reduction in efficacy could be caused by the modulation of oligosaccharide attachment sites such as that identified at residue N370 which potentially results in the blocking of antibody-specific epitopes (6). For immunodeficient patients or individuals in the acute disease phase, drugs that block the viral infection are needed. Ideally, such agents would act early in the virus life cycle to decrease the damage done to the patient. The recent approval of SARS-CoV-2 protease inhibitors, such as Paxlovid, has shown the efficacy of employing agents that target the early aspects of the viral replication cycle (7). Additional agents, capable of inhibiting viral entry, would be excellent candidates to further expand the clinically useful repertoire of antiviral drugs against coronavirus infections.

**Fig. 1.**
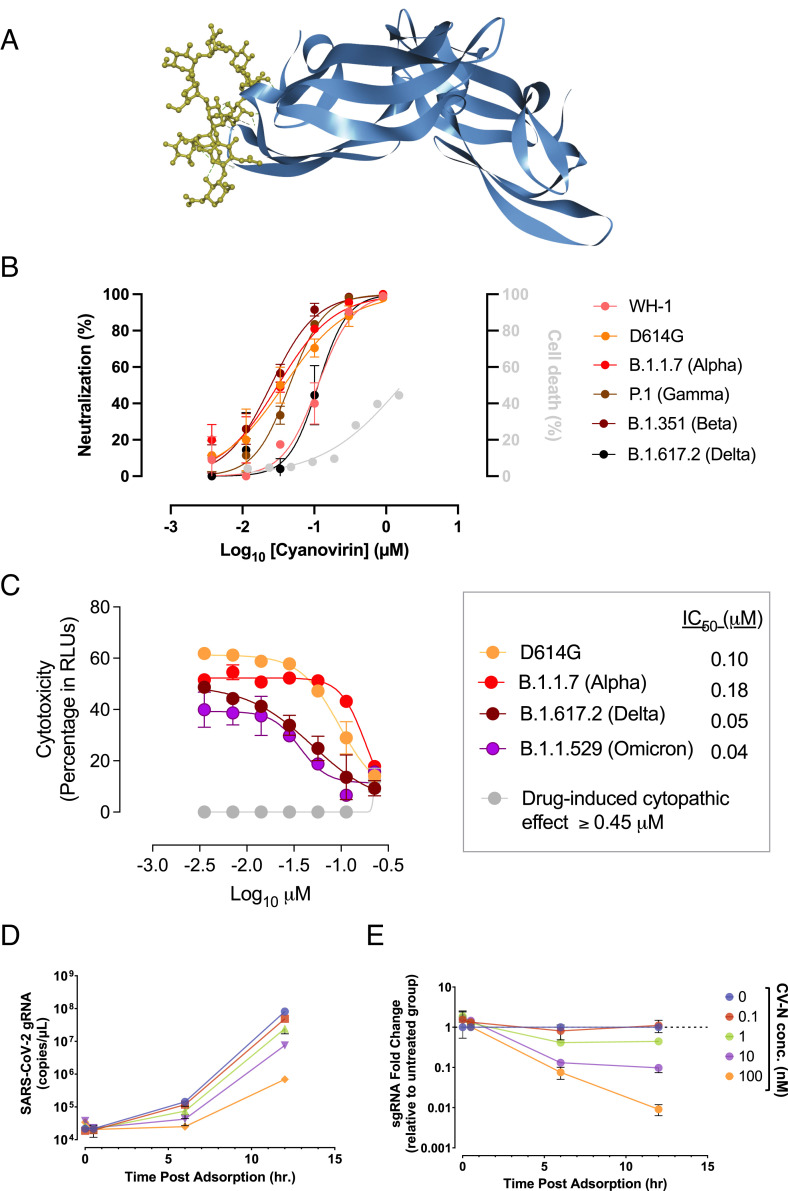
Cyanovirin-N shows activity against SARS-CoV-2 variants. (*A*) Interaction between CV-N (blue) and a high-mannose oligosaccharide (yellow). (*B*) Initial data showing inhibition of SARS-CoV-2 pseudovirus infection by CV-N. Colored circles indicate pseudovirus inhibition of various strains of SARS-CoV-2; (*C*) viral-induced cytopathic effect on Vero E6 cells exposed to different SARS-CoV-2 VOC in the presence of increasing concentrations of CV-N. Nonlinear fit to a variable response curve showing mean values and SEM from two representative experiments with four replicates are shown, excluding data from drug concentrations with associated toxicity. The IC_50_ value for each VOC is indicated. Cytotoxic effect on Vero E6 cells exposed to increasing concentrations of CV-N in the absence of virus is shown (gray lines) in relative light units (RLUs). Colored circles indicate various strains of SARS-CoV-2; (*D*) Time progression of SARS-CoV-2 genomic RNA count from Vero E6 hACE-2/hTMPRSS2 infected with OmiSP isolate pre-treated with various CV-N concentrations; (*E*) Subgenomic RNA of N gene transcription relative to untreated controls at different time points after viral adsorption. Data plotted as Mean ± SD of three independent experiments.

One promising class of viral entry inhibitors is the carbohydrate-binding proteins, known as lectins, which can inactivate a broad range of viruses by binding to the glycan structures present on the virus surface and inhibiting viral entry (8). A previous study of 33 plant-derived lectins revealed that many showed activity against SARS-CoV-1, with EC_50_ values in the mid-nanomolar range (9). Cyanobacterial lectins such as cyanovirin-N (CV-N) and scytovirin have been shown to target oligosaccharides that decorate viral envelope proteins on several viruses (10–14). The lectin CV-N which selectively targets high-mannose oligosaccharides (Fig. 1*A*) has been shown to be an entry inhibitor against multiple viruses including HIV (11), Ebola (12), influenza (13), and hepatitis C (14). Structurally distinct but mechanistically similar mannose-specific lectins have been shown to be active against the coronaviruses responsible for the original SARS outbreak (SARS-CoV-1) (15) and the subsequent outbreak of Middle East respiratory syndrome (16). The surface-exposed Spike protein of SARS-CoV-1 and SARS-CoV-2 is highly conserved (17, 18), each featuring multiple glycan structures with complex or high-mannose configurations (19), suggesting that CV-N, which has not yet been tested against coronaviruses, would be a good candidate for testing against SARS-CoV-2.

## Results

### CV-N Inhibits Viral Entry in SARS-CoV-2 Pseudovirus Assays and Decreases the Viral-Induced Cytopathic Effect in Replication Competent Viruses.

To test if CV-N might interfere with SARS-CoV-2 entry, first we tested *Escherichia* *coli*-produced CV-N for the ability to inhibit SARS-CoV-2 pseudovirus infection of human ACE2^+^ HEK 293 cells. Initial results against the Wuhan Strain (WH-1) of SARS-CoV-2 indicated that CV-N partially inhibited pseudovirus infection with an EC_50_ of ~100 nM (*SI Appendix*, Fig. S1). Further studies of the ability of CV-N to inhibit SARS-CoV-2 pseudoviral entry were conducted with six different Spike (S) proteins from variants of SARS-Co-V-2 (WH-1, D614G, B1.1.7 (Alpha), P.1 (Gamma), B1.351 (Beta), and B.1.617.2 (Delta)) showed that CV-N retained activity against all of the variants, with EC_50_ values between 31 and 112 nM (Fig. 1*B*). CV-N showed toxicity against the VeroE6 cells at higher test concentrations (>1 µM) slightly lower than that reported previously in Main-Darby canine kidney (MDCK) cells (13). Additional studies were conducted after the appearance of the Omicron strain of SARS-CoV-2 comparing the activity of CV-N against WH-1, Delta and Omicron variants in a live virus-induced cytopathicity assay. Similar to prior results using pseudoviruses, CV-N was active against all tested variants with EC_50_ values ranging from 40 nM for Omicron to 180 nM for Alpha (Fig. 1*C*). CV-N was well tolerated by Vero E6 cells until concentrations of >0.45 µM (Fig. 1*C*, gray circles). Importantly, CV-N was shown to be most active against the Delta and Omicron variants with EC_50_ values <50 nM, indicating increased sensitivity to CV-N for more recent SARS-CoV-2 variants. In addition to both the pseudovirus and cytopathicity assays, we conducted a SARS-CoV-2 Omicron virus adsorption assay to determine if CV-N reduced viral titers in cell cultures. As observed in Fig. 1 at early time points such as 0 and 0.5 h post adsorption, genomic RNA (gRNA) copies (Fig. 1*D*) and subgenomic RNA relative expression (Fig. 1*E*) are similar to the untreated control, suggesting that CV-N does not inhibit SARS-CoV-2 viral particle attachment to cell surface. At late time points, the level of gRNA copies and sgRNA relative expression drops in a dose-dependent manner, with the most dramatic difference in viruses treated with 100 nM of CV-N (~100-fold relative to untreated control), suggesting that CV-N inhibits SARS-CoV-2 entry by blocking viral envelope fusion.

### CV-N Is Active Against Replicating Strains of SARS-CoV-2 in a Plaque Reduction Assay.

To further address the inhibitory activity of CV-N against SARS-CoV-2, a plaque reduction assay was performed in Vero cells with nine different SARS-CoV-2 isolates representative of early and currently circulating variants in Brazil (*SI Appendix*, Table S1). As in the pseudovirus assay, CV-N was able to reduce the infectivity of all tested SARS-CoV-2 strains but showed differential activity depending on the viral strain, with the highest IC_50_ of 89.8 nM for 4,117 isolate (B.1.1.33 lineage, circulating in Southeast Brazil in 2020) and the lowest value of 0.674 nM for the emerging Omicron variant (Table 1 and *SI Appendix*, Fig. S2). All VOC tested herein (Gamma, Delta and Omicron) and one variant of interest (Zeta) were successfully neutralized by CV-N, with the Omicron strain having the greatest sensitivity, in accordance with the results of the antiviral assay (Fig. 1*C*).

**Table 1.** Activity of CV-N against patient isolates of SARS-CoV-2 in plaque reduction assays

SARS-CoV-2 isolate	Pango variant^*^	WHO variant^†^	IC_50_ (95% CI^‡^, [nM])	IC_90_ [nM]
SP1	B	–	8.353 (6.753-10.33)	109.65
RJ1	A.2	–	9.937 (8.177-12.08)	114.82
RJ2	B.1.1.33	–	14.86 (10.85-20.36)	158.49
4117	B.1.1.33	–	89.77 (76.85-104.9)	549.54
6439	B.1.1.33	–	13.03 (9.246-18.36)	269.15
814	P.2	Zeta	6.685 (4.836-9.241)	208.93
P1USP	P.1	Gamma	33.76 (27.56-41.36)	218.78
49947	B.1.617.2	Delta	7.291(4.914-10.82)	109.65
OmiSP	B.1.1.529	Omicron	0.674 (0.5271-0.8607)	6.46

^*^Data generated by https://pangolin.cog-uk.io/.

^†^Nomenclature stated by World Health Organization, https://www.who.int/en/activities/tracking-SARS-CoV-2-variants/.

^‡^95% CI of CV-N IC_50_ estimation.

### CV-N Binds to SARS-CoV-2 Spike but not to the Receptor Binding Domain (RBD).

**Fig. 2.**
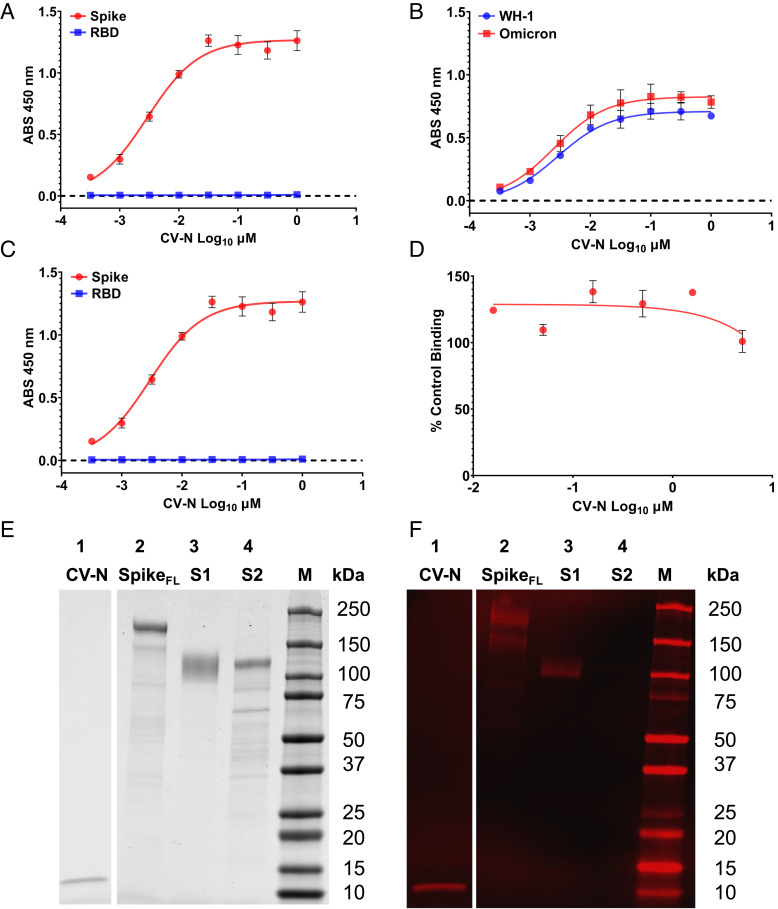
Cyanovirin-N binds to SARS-CoV-2 Spike glycoprotein but not to the isolated receptor binding domain of Spike. (*A*) CV-N binds to full-length SARS-CoV-2 spike glycoprotein (red) but not to the isolated receptor binding domain (RBD) of Spike (WH-1) (blue). (*B*) CV-N binds to Spike of both WH-1 (blue) and Omicron (red) variants. (*C*) ACE2 binding to both SARS-CoV-RBD (Blue) and full-length Spike (WH-1) glycoprotein (red). (*D*) CV-N binding to Spike (WH-1) does not inhibit the subsequent binding of ACE2. (*E*) Coomassie blue-stained SDS-PAGE gel showing protein bands of Spike (WH-1) glycoproteins blotted to PVDF membrane and (*F*) western blot results showing binding of CV-N to the S1 domain of SARS-CoV-2 Spike: western blot after binding to CV-N and visualization (in red) with rabbit polyclonal anti-CV-N antibodies. Lane 1: CV-N control, Lane 2: full-length SARS-CoV-2 Spike, Lane 3: S1 domain of SARS-CoV-2 Spike, Lane 4: S2 domain of SARS-CoV-2 Spike, M: Molecular weight standards.

After the demonstration of broad-spectrum activity of CV-N against SARS-CoV-2, multiple enzyme-linked immunosorbent assay (ELISA) experiments were conducted to examine the binding of CV-N to the Spike glycoprotein of SARS-CoV-2. Fig. 2*A* shows the binding of CV-N to Spike at low CV-N concentrations (EC_50_ = 2.9 nM). The same experiment sought to detect the binding of CV-N to the isolated receptor binding domain (RBD) of Spike. The results indicated no significant binding to RBD (Fig. 2*A*). CV-N binding to the original WH-1 variant Spike protein was also compared to CV-N binding to Omicron Spike. As seen in Fig. 2*B*, CV-N bound to Omicron Spike protein slightly better than to the WH-1 Spike protein. To put CV-N’s binding to Spike, and lack of binding to RBD, into context, we also tested the binding of ACE2 to both Spike and RBD. As depicted in Fig. 2*C*, ACE2 bound to both the isolated RBD (EC_50_ = 63.9 nM) and Spike (EC_50_ = 25.9 nM). Importantly this latter binding interaction was shown to be weaker than CV-N’s binding to Spike, highlighting the potential of CV-N to out compete ACE2 for binding the viral glycoprotein. Finally, as CV-N bound Spike with significantly greater affinity than ACE2, we tested CV-N’s ability to block subsequent ACE2 binding to Spike. As depicted in Fig. 2*D*, CV-N did not significantly inhibit ACE2 binding to Spike at concentrations up to 5 µM. This result mirrors both the results of our SARS-CoV-2 viral adsorption assay (Fig. 1 *D* and *E*) and previous studies with HIV-1 gp120 wherein CV-N binding to gp120 did not inhibit the subsequent binding of sCD4 to gp120 (11).

### CV-N Binds to Isolated S1 Domain of SARS-CoV-2 Spike.

CV-N bound with good affinity to Spike protein and did not bind to the RBD. We therefore sought to further define the binding region of the Spike protein which binds to CV-N. SARS-CoV-2 Spike protein differs from the Spike protein from SARS-CoV-1 in that it exhibits a furin-cleavage site, which is readily processed upon production by cells, resulting in a S1 (AAs 1 to 614) with the RBD and S2 domain (AAs 615 to 1273) which are responsible for the fusogenic activity of Spike (20). Recombinantly produced full-length Spike protein, which we used in both ELISA and isothermal titration calorimetry (ITC) experiments, contains a mutation that abolishes the furin-cleavage site to allow the production of full-length protein. We therefore evaluated the binding of CV-N to S1 and S2 domains of SARS-CoV-2 Spike protein by sodium dodecyl sulfate polyacrylamide gel electrophoresis (SDS-PAGE) and western blot analysis using isolated S1 and S2 domains produced in HEK293 cells (Fig. 2 *E* and *F*). The stained SDS-PAGE result. The stained SDS-PAGE result (Fig. 2*E*) is included to show the level of purity used in the western blot analysis. We show that CV-N selectively binds to full-length Spike protein and the isolated S1 domain of Spike (Lanes 2 & 3, respectively of Fig. 2*F*) but not to the S2 domain of SARS-CoV-2 Spike protein (Lane 4, Fig. 2*F*).

### CV-N Binding Affinity is Higher for Omicron Spike than Previous Variants.

**Fig. 3.**
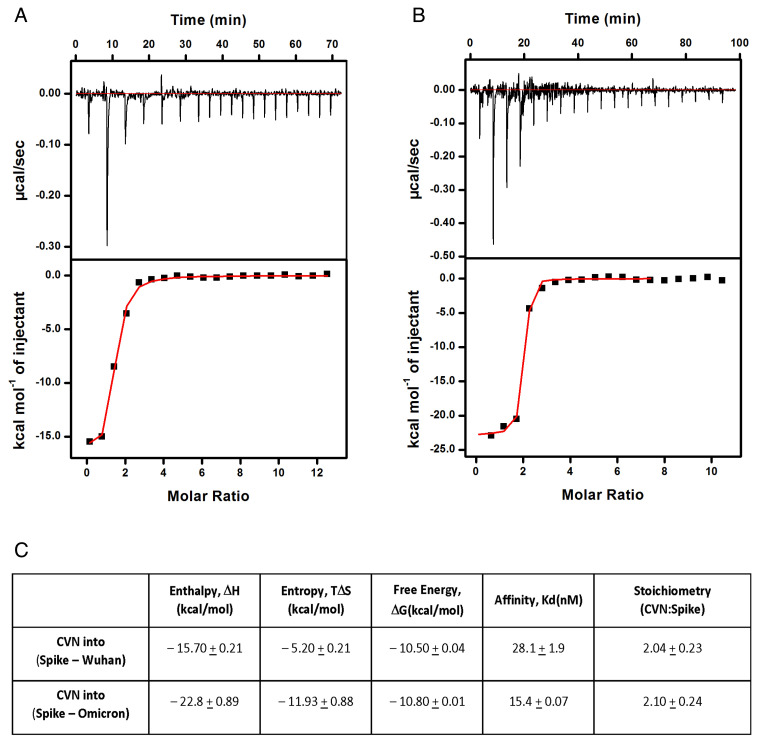
Cyanovirin-N binds to both Wuhan and Omicron SARS-CoV-2 Spike glycoprotein with a stoichiometry of 2 cyanovirin-N:1 Spike monomer. Isothermal titration calorimetry data showing the binding of CV-N to SARS-CoV-2 Spike glycoprotein. (*A*) Thermogram and calculate binding to WH-1 variant Spike. (*B*) Thermogram and calculate binding to Omicron variant Spike. (*C*) Calculated energies of interaction, affinities, and stoichiometry for CV-N and both WH-1 and omicron Spike glycoproteins. Spike glycoproteins were both produced in HEK293 cells.

The binding interaction between CV-N and Spike (WH-1) protein was next quantified using isothermal titration calorimetry (ITC) (Fig. 3 *A* and *B*). ITC provides the full thermodynamics of the binding, a defined binding constant, and the stoichiometry of interaction of the binding partners. For these ITC studies, we initially used SARS-CoV-2 S protein produced in two expression systems, the baculovirus and mammalian HEK293 expression systems. This was done in an effort to normalize our current and future studies of HEK293-produced glycosylated proteins against our previous studies with SARS-CoV-1 (15) where only the baculovirus-produced S protein was studied. CV-N binding to the baculovirus-produced trimeric Spike did show stronger binding (K_d_ = 13.4 nM) (*SI Appendix*, Table S2) than to trimeric HEK293-produced Spike protein (K_d_ = 28.1 nM; Fig. 3*C*), and this was presumably due to the fact that baculovirus-produced Spike likely presents more high-mannose oligosaccharides on its protein surface than the mixed oligosaccharide profile produced in human HEK293 cells (21) as CV-N is known to bind exclusively to high-mannose oligosaccharides (22). It is important to note here that recent publications have reported that that HEK293 cell-produced Spike (WH-1) protein contained a higher amount of complex oligosaccharides in comparison with Vero E6 cell-produced Spike (WH-1) which contained predominantly high-mannose glycans (23). Thus, the affinity of CV-N to HEK293-produced Spike is likely also lower than that produced in Vero E6 cells. The stoichiometry of interaction of ~2.4 CV-N per monomer of baculovirus-produced Spike (*SI Appendix*, Table S2) also supported this conclusion in that CV-N was able to recognize an additional site on Spike produced in baculovirus (and potentially in Vero E6 cells). For all subsequent experiments, we used only HEK293-expressed Spike proteins.

A comparison of CV-N binding to the WH-1 versus the Omicron Spike protein (Fig. 3*C*) showed stronger affinity for the latter, more recent variant of the SARS-CoV-2 Spike. The enthalpy of binding to Omicron Spike protein was more exothermic suggesting an increase in the number of polar/electrostatic contacts occurred in this interaction compared to the WH-1 Spike. This increase may have been responsible for the improved binding affinity of CV-N with Omicron Spike. Both binding experiments resulted in a calculated stoichiometry of two CV-N bound per Spike monomer, suggesting that CV-N bound to the same number of sites on the respective Spike proteins. Nevertheless, its overall set of binding contacts (polar/electrostatic, hydrogen, and van der Waals) at these sites was greatly improved with the Omicron Spike (Fig. 3*C*), resulting in an affinity almost twice as strong (K_d_ = 15.4 nM) than for the CV-N:WH-1 Spike (K_d_ = 28.1 nM)_._ Not surprisingly, an enthalpy-entropy compensation was also seen for the Omicron Spike, with a decreased entropy of interaction indicating that the bound complex likely occupied a more “fixed” state that disallowed favorable entropic contributions (rotational, vibrational, and hydration) to the binding. This enthalpy-entropy compensation resulted in an overall free energy of the interaction (ΔG) that was similar to what was observed in the WH-1 Spike experiment.

### CV-N Binds to Three Oligosaccharide Attachment Sites of SARS-CoV-2 Spike S1 Domain.

Western blot data had indicated that CV-N bound exclusively to the S1 domain of the Spike protein (Fig. 2*F*) but did not appear to bind to the RBD within S1 (Fig. 2*A*). In addition, ITC data had resulted in the determination that two CV-N molecules bound per monomeric unit of Spike protein. We therefore sought to define at which sites within the S1 region CV-N bound. CV-N is known to target specific oligosaccharides on envelope glycoproteins (22), so we concentrated on mapping to which oligosaccharides CV-N might bind. The SARS-CoV-2 Spike protein from the WH-1 variant has been reported to contain 22 oligosaccharide attachment sites, all of which are glycosylated (24). Of these, ~30% have been determined to bear oligomannosides to which CV-N might bind. Within the S1 domain, there are reported to be ten N-linked oligosaccharide attachment sites, two of which are found in the RBD. The RBD oligosaccharide attachment sites have been determined to bear predominantly complex oligosaccharides (24). CV-N was previously shown not to bind to complex oligosaccharides (22), so its lack of binding to the isolated RBD domain is consistent with expectations based on the published literature. Of the remaining eight N-linked oligosaccharide attachment sites present in the S1 domain, four were reported to bear primarily oligomannosides, and these are found at positions N61, N122, N234, and N603 (24).

**Fig. 4.**
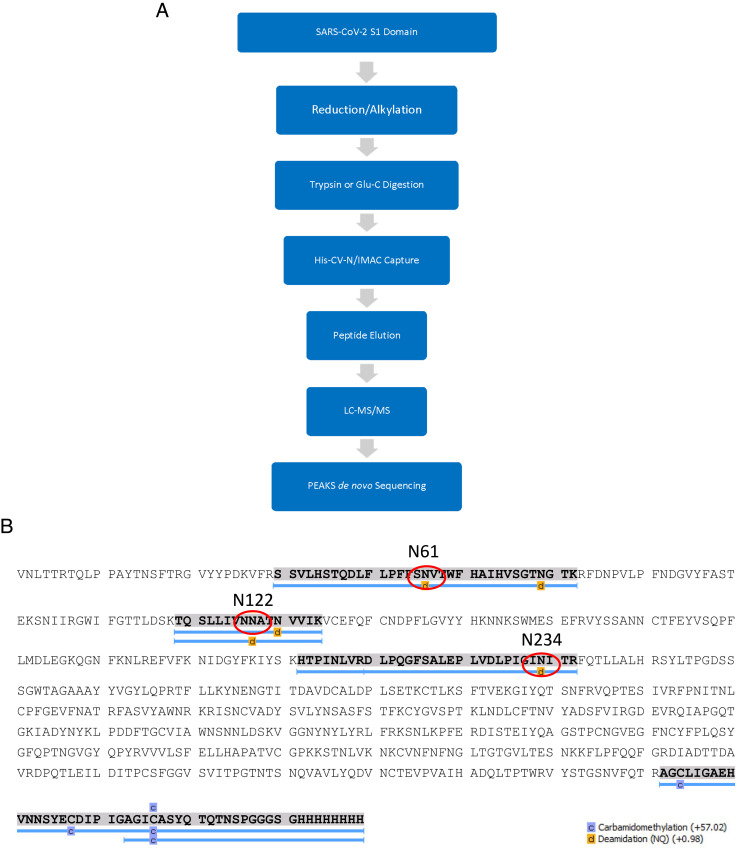
Determination of potential cyanovirin-N binding sites on the S1 domain of SARS-CoV-2 Spike glycoprotein. (*A*) Schematic of experimental procedure. (*B*) Amino acid sequence of S1 domain of SARS-CoV-2 Spike S1 domain (WH-1) showing peptide coverage and specific oligosaccharide attachment sites of CV-N binding S1 peptides.

To identify which of these residues CV-N might bind, we used a CV-N affinity column and SARS-CoV-2 S1 domain proteolytic digests to capture specific peptides which bound to CV-N. The resulting peptides were separated by liquid chromatography tandem mass spectrometry (LC/MS-MS) and sequenced to identify the regions of the S1 domain bound by CV- N (schematic of procedure shown in Fig. 4*A*). Our results indicated that the CV-N affinity column appeared to selectively bind to S1 digest peptides that included N-linked oligosaccharide attachments sites at residues N61, N122, and N234 (Fig. 4*B*). The peptide containing oligosaccharide attachment sites N61 and N71 were a single peptide as the proteases did not cleave between these two residues (Fig. 4*B*). The C-terminal peptide of the S1 domain, which does not bear any oligosaccharides, was captured by the affinity column via its His-tag. Oligosaccharide attachment sites are known to be differentially populated (25), and as such, our results indicate that CV-N can, on average, be bound to two of these three oligosaccharide attachment sites on the S1 domain of SARS-CoV-2.

### Modeling of CV-N Bound to Oligosaccharides on SARS-CoV-2.

**Fig. 5.**
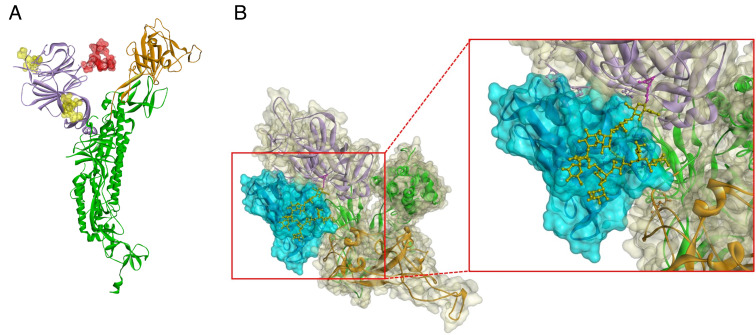
Molecular model of cyanovirin-N bound to N234 high-mannose oligosaccharide on SARS-CoV-2 Spike. (*A*) Omicron Spike glycoprotein monomer with glycans at N61, N122 (yellow), and 234 (red). Interaction between CV-N (blue surface) and S1 (white surface and green structure). Mannose oligosaccharide (N234) is shown in ball and sticks (yellow). The NTD is shown in purple and the RBD in orange. (*B*) Interaction between CV-N (blue surface) and S1 (light yellow surface). Mannose oligosaccharide (N234) is shown in ball and sticks (yellow). The NTD structure is shown in purple and the RBD structure in orange. The N234, shown in pink, is the residue who bound the mannose oligosaccharide to the S1 structure.

To visualize the binding of CV-N to SARS-CoV-2 Spike protein, molecular modeling was performed with Omicron Spike protein while constricting the model based on the ITC-determined stoichiometry of CV-N for Spike and the identified oligosaccharide attachments sites on S protein identified by the CV-N affinity column experiments. Only the three attached oligosaccharides experimentally identified as binding to CV-N are shown decorating the S1 region of the Spike glycoprotein (N61, N122 & N234, Fig. 5*A*). Fig. 5*B* shows a close-up depiction of the model of CV-N bound to the high-mannose oligosaccharide at position N234 which has previously been reported to play an important role in viral infectivity (26). The model supports both the specific binding of CV-N to the S1 domain of Spike while showing that CV-N does it sterically block the RBD interface responsible for binding ACE2. This is supported by ELISA studies showing that ACE2 binding to Spike is not significantly inhibited by CV-N (Fig. 2*D*) and subsequent viral adsorption studies that show live virus binding to ACE2^+^ target cells is not inhibited by CV-N pre-treatment (Fig. 1 *D* and *E*).

### Activity of Intranasal (i.n.) CV-N against SARS-CoV-2 in a Hamster Model System.

To validate the inhibitory activity of CV-N against SARS-CoV-2 observed in vitro assays, we tested the efficacy of CV-N intranasal administration in Syrian golden hamster model for SARS-CoV-2 infection. Previous in vivo studies done with mice showed that doses above 5 mg/kg/d of CV-N are toxic by intranasal (i.n.) delivery (12). Therefore, a lower dosage regimen was selected for a hamster challenge model. Animals were administered either 0.04, 0.2, or 1.0 mg/kg of CV-N twice daily, starting on Day-1 (24 h prior to virus inoculation) and continuing for 4 d (*SI Appendix*, Table S3). A matching minimalessential medium (MEM) control arm was also included. Animals were challenged with the 2 × 10^5^ plaqueforming units (PFU) of WH-1 variant of SARS-CoV-2 on day 0, were monitored for body weight, and were euthanized on Day 7 to measure SARS-CoV-2 RNA copies in lung homogenate. In addition, histologic evaluation of lung tissues was undertaken. The results showed variable effects on body weight changes with only female hamsters showing significant protection from weight loss when administered 0.2 mg/kg/d CV-N (*SI Appendix*, Fig. S3 *A*–*C*). Male hamsters did not show a similar positive effect on weight loss. The high dose arm of 1.0 mg/kg/d displayed the most significant negative effects on weight which we ascribe to potential CV-N-induced toxicity. CV-N treatment did result in a significant reduction in infiltrates in hamster lungs as depicted by hematoxylin/eosin (H & E) staining of control and treated lung tissues (*SI Appendix*, Fig. S3 *D* and *E*). Based on these preliminary experiments, we designed a new treatment protocol in which 12 animals were treated (six female and six males) once with a single high dose of CV-N (2 mg/kg) and immediately challenged with 2 × 10^5^ PFU of WH-1 variant of SARS-CoV-2. A sham group of 12 additional animals (same proportion of males and females) were treated with phosphate buffered saline (PBS) and challenged the same way. The animals were monitored for body weight, and six animals from each group were euthanized on Day 4 and Day 7 to measure SARS-CoV-2 RNA copies in lung homogenates. In addition, histologic evaluation of lung tissues was undertaken.

**Fig. 6.**
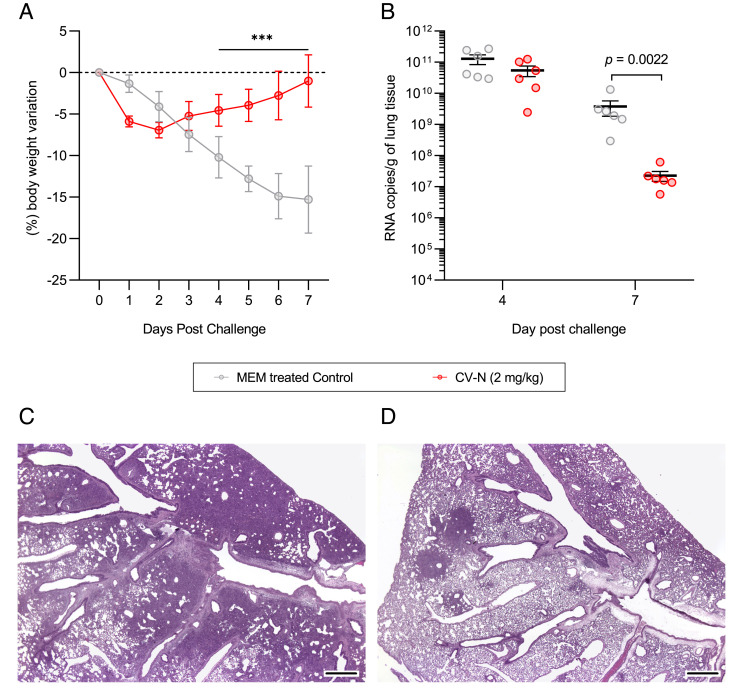
Results of intranasal administration of cyanovirin-N on SARS-CoV-2 infection in Syrian golden hamster model. Intranasal administration of 2 mg/kg CV-N reduces both (*A*) weight loss and (*B*) viral RNA copies in lungs of treated animals in a Syrian golden hamster model for SARS-CoV-2 infection. Images (1.25×) of tissue damage in both control (*C*) and CV-N treated (*D*) hamsters. Darkened basophilic areas (dark purple) are widespread in control animals (*C*), whereas they are less severe and multifocal in CV-N treated hamster lungs (*D*). (Scale bars indicate 100 µm).

The single-dose treatment with CV-N improved weight recovery by Day 3 in comparison with sham-treated group (Fig. 6*A*). This result was consistent in both male and females (*SI Appendix*, Fig. S4 *A* and *B*). Also, there was a significant reduction of RNA copies count in lungs by Day 7 in CV-N treated group as determined by PCR detection of genomic viral RNA (>3 log, Fig. 6*B*). This data was in accordance with histopathologic findings in lung tissue slides, as is shown in *SI Appendix*, Fig. S6 *C* and *D* (evidenced by a reduction in darkened basophilic areas in treated animals), where the lungs of control animals were shown to be heavily infiltrated with immune cells and contained limited alveolar space consistent with SARS-CoV-2 infection (Fig. 6*C*, dark purple areas), while bronchiolo-alveolar hyperplasia and syncytial cells were decreased in severity and/or incidence in CV-N-treated animals (2 mg/kg CV-N, Fig. 6*D*). The data demonstrate a noticeable protective effect of CV-N on the lungs of SARS-CoV-2 challenged animals.

## Discussion

We have previously reported that the antiviral lectin CV-N displayed potent activity against HIV (11), Ebola (12), hepatitis C (14), and influenza (13). It has been shown that CV-N inhibits entry by such viruses through specific binding to oligomannoside structures (Fig. 1*A*) decorating the surface glycoproteins of these enveloped viruses (22, 27). With the COVID-19 pandemic in 2020 (28, 29), we established an international collaboration to assess the potential utility of CV-N against this enveloped coronavirus. Initial in vitro results in both the USA and Spain indicated that CV-N was active at low-moderate nanomolar concentrations against a variety of SARS-CoV-2 strains and variants when tested in pseudovirus and live virus assay systems (Fig. 1 *B* and *C*). Against patient isolates from Brazil (30), CV-N inhibited the cytopathic effects of SARS-CoV-2 (Table 1 and *SI Appendix*, Fig. S2). Although all isolates were successfully neutralized by CV-N, the tested SARS-CoV-2 strains showed a wide spectrum of sensitivity to CV-N, with IC_50_ values ranging from ~0.7 to 90 nM (Table 1). The Omicron strain was the most susceptible to CV-N, despite the significant amino acid substitutions in the Spike protein. Importantly, as Spike oligomannosides are the primary CV-N target, alterations specifically on glycosylated amino acids such as asparagine residues could explain this diversity in CV-N sensitivity by different SARS-CoV-2 strains. However, none of the Spike amino acid substitutions described herein (*SI Appendix*, Table S1) appear to impact directly in gain or loss of glycosylation sites. These data suggest that glycosylated sites on Spike are conserved, and this could imply an advantage for the use of antiviral lectins, such as CV-N, against COVID-19 infections. Taken together, these in vitro results were consistent with previously published reports on the activity of oligomannoside-specific antiviral lectins against members of the *Coronaviridae* family (15) and suggest that CV-N inhibits viral entry, acting directly on SARS-CoV-2 viral particles. Previous studies on CV-N demonstrated that it binds to surface envelope glycoproteins on viruses and prevents viral entry (22, 27, 31, 32). To confirm the molecular target of CV-N against SARS-CoV-2, we undertook a number of binding studies. ELISA studies showed that CV-N bound to the SARS-CoV-2 Spike glycoprotein but did not bind isolated receptor binding domain (RBD) of the Spike glycoprotein (Fig. 2*A*). Previously on in silico studies of the binding of CV-N to non-glycosylated RBD which reported that CV-N bound to RBD (33) differs from our results here and the previously-understood binding parameters of CV-N which show that binding is dependent on glycosylation. Additional in vitro studies in that report with glycosylated glycoproteins mirror our findings that CV-N binds to the S1 domain of Spike and inhibits viral infectivity. Further studies to further assess any potential functional signal of RBD binding by CV-N showed that CV-N did not inhibit the binding of Spike to the human cellular target of SARS-CoV-2 Spike, ACE2 (Fig. 2*D*). This result mirrors results previously published for the binding of CV-N to HIV-1 gp120 which showed that, though CV-N bound to gp120, it did not significantly block subsequent engagement with sCD4 (11, 34). The current results and previously published reports on other enveloped viruses suggest that CV-N is acting as an entry inhibitor of SARS-CoV-2 rather than as an inhibitor of viral attachment. Data generated for this manuscript with SARS-CoV-2 Omicron in a viral adsorption assay that showed no inhibition of viral particle attachment to ACE2^+^ cells following CV-N pre-treatment provided additional evidence for this likely mechanism of action.

We further evaluated the direct binding of CV-N to the SARS-CoV-2 Omicron Spike glycoprotein. ELISA studies indicated that CV-N bound slightly better to the Omicron Spike when compared to that from the original WH-1 variant (Fig. 2*B*). We also used ITC to determine the affinity and stoichiometry of CV-N for Spike variants produced in both baculovirus and HEK293 expression systems. These expression systems (as well as the VeroE6 cells used for in vitro assays) have varying abilities to decorate envelope glycoproteins with high-mannose oligosaccharides to which CV-N binds (23, 35). This can alter the measurable affinity of CV-N (or other lectins) for envelope glycoproteins. CV-N was found to bind to both Spike variants produced in HEK293 cells with a stoichiometry of two CV-N per Spike monomer (Fig. 3 *A−C*) indicating multiple binding sites of CV-N on Spike. Importantly, as shown in Fig. 3*C*, the affinity of CV-N for Omicron Spike appeared to be almost twice that shown for WH-1 Spike with Kd values of 15.4 nM and 28.1 nM, respectively. The affinity of CV-N for SARS-CoV-2 Spike was slightly greater than previously reported for CV-N to HIV-1 gp120 (Kd = 37 nM) produced in mammalian cells (32). The lack of engagement of RBD by CV-N, and its greater affinity for mutated Spike glycoproteins, is important for its potential use against SARS-CoV-2 as many of the reported resistance mutations in Spike, that reduce the efficacy of both vaccines and monoclonal antibody therapies (36, 37), are found in the RBD. Our results provide an indication that the mechanism of action of, and mechanisms for resistance to, CV-N is distinct from these immunologic agents.

As CV-N bound to SARS-CoV-2 Spike with a 2:1 stoichiometry and high affinity, but did not bind to the RBD, we sought to further define the binding interaction between these two proteins. Initial studies centered on the major regions of Spike, S1 and S2, which are separated by furin cleavage during glycoprotein processing by the host cell (20). In western blot experiments, we determined that CV-N bound only to the S1 domain of Spike (Fig. 2*F*). The S1 domain (AA1-AA681) contains both the N-terminal domain (AA13-AA305) and the RBD (AA319-AA541) (20). Having already determined that CV-N did not bind to the RBD, we concentrated our efforts on deducing where CV-N might bind within the rest of the S1 domain. Previous studies on the glycosylation of SARS-CoV-2 Spike showed that Spike monomer bore 22 possible N-linked oligosaccharide attachment sites (24). Of these, 11 were found in the S1 domain. Seven of these sites were found to be decorated with predominantly complex oligosaccharide structures known to bear terminal sialic acid residues (N17, N74, N149, N165, N282, N331, and N343) when produced in HEK293 cells. CV-N has been shown to selectively bind only to oligomannosides which have mannose residues on their termini (22, 31, 32) and so was unlikely to bind at any of these sites. The remaining four sites in the S1 domain, N61, N122, N234, and N603, were all reported to be primarily decorated with oligomannosides, with N234 being almost exclusively decorated with a high-mannose oligosaccharide (24). As oligosaccharide structures at specific residues are variable, only an average propensity for a specific glycan structure can be determined. We therefore hypothesized that CV-N was likely binding to an average of two of these four possible oligosaccharides, which then caused subsequent viral entry inhibition.

To test this hypothesis, we designed an experiment using a CV-N affinity column similar to that we previously reported (27) to capture peptides bearing oligosaccharides that could be bound by CV-N (Fig. 4 *A* and *B*). The results indicated that CV-N bound only to peptides decorated with oligomannosides and further refined the CV-N binding sites within the N-terminal domain (NTD) of Spike. This result confirmed our earlier ELISA studies showing that CV-N did not bind to the RBD of Spike as the peptide containing the two oligosaccharide attachment sites in RBD at positions N331 and N343 was not captured by the CV-N affinity column. It also matched expectations for the specificity of CV-N binding to oligosaccharides as the six predominantly complex oligosaccharide-bearing sites were also not retained by the CV-N column. Only that at N74, which was not separated from the oligomannoside-bearing N61, was retained.

The potential functional significance of CV-N binding of oligomannosides at positions 61, 122, and 234 within the NTD of SARS-CoV-2 Spike is still not fully understood. Fig. 5*A* shows the location of these oligosaccharides on SARS-CoV-2 Spike from the Omicron variant. As CV-N does not inhibit the binding of Spike to ACE2, it is unlikely that the binding of CV-N to any of these oligomannosides on Spike sterically inhibits viral attachment. Recent reports have, however, suggested an important role for the high-mannose oligosaccharide at N234 of Spike. An initial report indicated that mutation of the N-linked oligosaccharide attachment site at N234 to remove the possibility of oligosaccharide attachment reduced viral infectivity by 40% (26). Subsequent cryo-EM studies determined that the glycan at position N234 played important roles in both shielding the RBD and in stabilizing the RBD in the “up” conformation (38). Fig. 5*B* depicts a model of CV-N bound to the predominantly high-mannose oligosaccharide at N234 with a close-up view of the modeled binding interface. Though suggestive of a potential mechanism of action for CV-N, the role of the oligosaccharide at N234 has been suggested to be important for viral attachment, which is not inhibited by CV-N. Further studies will be necessary to show the functional consequences of CV-N binding to oligosaccharides on SARS-CoV-2 Spike, including at position N234, N61, and N122.

Intranasal administration of CV-N has previously been shown to be effective against influenza infection in mice, with broad-spectrum activity against both influenza A and B (13). Here, we assessed CV-N efficacy in a Syrian golden hamster model of SARS-CoV-2. Different daily-administered doses were tested in a pilot experiment (0.04, 0.2, and 1.0 mg/kg/d) (*SI Appendix*, Table S3). In this assay, we observed some protection against weight loss, but only in male hamsters (*SI Appendix*, Fig. S3 *A*–*C*). However, the highest CV-N daily dose of 1.0 mg/kg/d showed toxicity, with premature body weight loss of treated animals and a slight recovery by day 4. It has been reported that CV-N can induce differential toxicity depending on the administration route. Earlier reports indicated that repeated intranasal administration led to more toxic outcomes in mouse model than previously reported for the subcutaneous route (12, 13). To assess the prophylactic potential of CV-N against SARS-CoV-2, we tested another administration pre-treatment protocol where hamsters were treated via intranasal administration with a single dose (2.0 mg/kg) of CV-N prior to challenge with SARS-CoV-2 (*SI Appendix*, Table S4). This prophylactic protocol led to body weight recovery as soon as day 3 after viral challenge (Fig. 6*A*), with a 2-log reduction in viral RNA copies in lungs on day 7 (Fig. 6*B*) and significantly less tissue damage in lungs (Fig. 6 *C* and *D*), suggesting that CV-N is active against SARS-CoV-2. These results are in agreement with those previously published on the activity of another oligomannoside-specific antiviral lectin, griffithsin, against SARS-CoV-1 (15) which also showed evidence of antiviral efficacy. Intriguingly, though CV-N pre-treatment resulted in a reduction in viral titers in the lungs of hamsters on day 7, there was not a significant reduction apparent on day 4. This lack of apparent efficacy at day 4 is similar to that reported for the antiviral lectin griffithsin following intranasal administration in the mouse model system for SARS-CoV-1. In that pre-treatment model, griffithsin did not significantly reduce viral titers in lungs at early time points but did show significant reductions at day 7 (15). Why these lectins do not show the expected differences in viremia at early time points will require further, in depth studies, to fully understand. Similar data were also seen for another protein-based intranasal treatment of SARS-CoV-2 in animal model systems. Huo et al. (39) found that pre-challenge intranasal treatment with an anti-Spike RBD nanobody resulted in only modest changes in viral titer at day 2 and day 4 post-challenge with more significant changes on day 7 post-challenge. The ~3-log reduction in the viral titer on day 7 in hamster lungs following intranasal CV-N treatment is significant, as is the apparent reduction in damage to the lungs of test animals, and supportive of the opportunity broad-spectrum antiviral lectins may present for antiviral prophylaxis.

The findings presented herein enhance the body of knowledge about the potential use of antiviral lectins in the context of a viral pandemic and aim for the readiness in implementing such molecules for possible prophylactic approaches against SARS-CoV-2 and other viruses. Other lectins, or modified versions of CV-N, could prove to be better candidates for further development. The lectin griffithsin, for example, has been shown to be non-toxic as demonstrated by its successful progression into human clinical trials to prevent viral infections (40, 41). Further clinical development of intranasal formulations of non-toxic lectins and less toxic CV-N analogs, such as PEGylated versions of CV-N (42, 43), could be especially beneficial for pandemic preparedness due to their demonstrated broad-spectrum activity against both influenza viruses (44) and coronaviruses (15, 45), two main categories of pathogens of continuing international concern for future potential pandemics (46). In addition, as lectins appear to work via a mechanism distinct from current anti-SARS-CoV-2 agents, such as vaccines, mAb therapy, and approved antiviral drugs, they offer a method of antiviral prophylaxis that could be synergistic with these other approaches. As a number of lectins with broad antiviral activity including CV-N (40, 41, 44) have been successfully produced in plant expression systems, in some cases in sufficient yields to support clinical trials (45–47), the cost-effective production of lectins for use as broad-spectrum antiviral agents, especially for use in developing countries, is achievable.

## Materials and Methods

### Sources of Pure Lectins and Viral Proteins.

CV-N was produced in the Molecular Targets Program, Center for Cancer Research, National Cancer Institute (NCI). The SARS-CoV-2 S1 protein produced in baculovirus-infected insect cells was sourced from Sino Biological (catalog no. 40150-V08B1). His-tagged HEK293-produced trimeric Spike (S1+S2) was purchased from BPS Bioscience catalog #100728. HEK293-produced SARS-CoV-2 Spike glycoprotein isolated domains S1 and S2 were either by the Protein Expression Laboratory, Frederick National Laboratory for Cancer Research (S1) or purchased from Millipore Sigma catalog # AGX820 (S2).

### SARS-CoV and SARS-CoV-2 Pseudovirus-Neutralization Assay.

HEK-293T cells (ATCC repository) were maintained in Dulbecco’s modified Eagle’s medium (DMEM) with 10% fetal bovine serum, 100 IU/mL penicillin, and 100 μg/mL streptomycin (Thermo Fisher Scientific). HEK-293T cells overexpressing human ACE2 were a gift from Integral Molecular Company and maintained in DMEM as above, also containing 1 μg/mL puromycin (Thermo Fisher Scientific). Pseudoviruses were prepared as previously reported (48). The p24gag content of all viruses was quantified by ELISA (Perkin Elmer), and viruses were titrated in HEK-293T cells overexpressing ACE2 to test the antiviral effect of CV-N against SARS-CoV-2 pseudoviruses in duplicate. The inhibitory capacity of CV-N was assessed after 48 h using the EnSight Multimode Plate Reader and BriteLite Plus Luciferase reagent (PerkinElmer). The values were normalized, and the ID_50_ (reciprocal dilution inhibiting 50% of the infection) was calculated as previously described (49).

### Calculation of IC_50_ for CV-N Using Different SARS-CoV-2 VOC on Vero E6 Cells (Spain).

Biosafety Approval**.** The biologic biosafety committee of the Research Institute Germans Trias i Pujol approved the execution of SARS-CoV-2 experiments at the Biosafety Level 3 (BSL-3) laboratory of the Center for Bioimaging and Comparative Medicine (CSB-20-015-M3). SARS-CoV-2 viruses were isolated from a nasopharyngeal swab collected in March 2020 in Spain in Vero E6 cells as described (50). To determine the IC_50_, the indicated serial dilutions of CV-N were added to 60,000 Vero E6 cells per well in 96-well plates in duplicates to determine the viral-induced cytopathic effect 72 h later. The relative light units were normalized, and the IC_50_ was calculated by plotting and fitting the log of CV-N concentration vs. cytotoxicity to a four-parameter equation in Prism 9. In parallel, cells exposed to CV-N in the absence of virus were assayed to detect any possible drug-induced cytotoxic effect using CellTiter-Glo Luciferase reagent.

### Plaque Reduction Assay against Replicating SARS-CoV-2 Strains.

Experiments handling replicative competent viruses were performed in a Biosafety Level 3 (BSL-3) laboratory located in the Laboratório de Virologia Molecular, Universidade Federal do Rio de Janeiro (UFRJ, Brazil. Information about the SARS-CoV-2 strains is summarized in *SI Appendix*, Table S1. The assay was conducted similarly to the PRNT assay commonly used to assess antibody neutralization titers against a virus. Experiment readout was given by plaque forming units (PFU) count and normalization to the untreated control inoculum. Briefly, serial fivefold dilutions of CV-N (from 4.65 × 10^−3^ to 1.82 × 10^3^ nM, i.e., 20 to 5.12 × 10^−5^ µg/mL) were incubated with 50 to 200 PFUs of SARS-CoV-2 suspension for 1 h at 37 °C in the incubator. After incubation, CV-N:SARS-CoV-2 suspensions were added to Vero cells (ATCC CCL-81) confluent monolayers seeded in 12-well plates to allow viral adsorption for 1 h at 37 °C. After the adsorption step, inoculum was removed, and cells were grown in semisolid Alpha-MEM (1.25 % carboxymethylcellulose; 1.5 % fetal bovine serum; 1 % Pen-Strep in Alpha-MEM) for 3 to 4 d, depending on viral strain. After infection, 10 % formaldehyde solution was added onto semisolid medium and cells were fixed for 1 h at room temperature. Fixed Vero cells monolayers were then washed and stained with crystal violet solution (0.5 % w/v crystal violet; 20 % v/v ethanol) for 15 min to allow PFU visualization and quantitation. Normalized results were expressed by Mean ± SEM and analyzed by nonlinear regression (best-fit method) using GraphPad Prism 8 to calculate IC_50_ values. IC_50_ and Hill-slope values obtained were imputed to estimate IC_90_ values for each SARS-CoV-2 isolate tested using Graphpad QuickCalcs.

### ELISA Assays.

Purified, recombinant SARS-CoV-2 Spike protein WH-1 (BPS Bioscience #100728), Spike protein Omicron (Protein Expression Laboratory, FNLCR) or Spike receptor binding domain (WH-1, R&D Systems #10500-CV-100), each produced in HEK293 cells, were immobilized on high-binding ELISA plates (Greiner #655081). Plates were washed with 1× PBS pH 7.4, 0.05% Tween-20 (PBS-T) and blocked with a solution of 5% (w/v) bovine serum albumin (BSA, Fisher #BP9706-100) in 1× PBS pH 7.4 (PBS). For evaluating the binding of CV-N to Spike variants and RBD, plates were washed three times with phosphate buffered saline with 0.01% Tween-20 (PBS-T) and incubated with serial half-log dilutions of CV-N, diluted in PBS, for 1 h at RT. Plates were washed three times with PBS-T and incubated with rabbit anti-CV-N polyclonal antibodies (11) for 1 h at RT, then washed three times with PBS-T and incubated with goat anti-rabbit IgG-HRP conjugate (Thermo Fisher Scientific #31460) for 1 h at RT, again washed three times with PBS-T and developed using 1-Step Ultra TMB-ELISA solution (Thermo Fisher Scientific #34028). The HRP reaction was stopped with 1 M hydrochloric acid, and absorbance values at 450 nm were measured on a SpectraMax i3x plate reader (Molecular Devices). For ACE2–Spike and RBD binding experiments, wells were incubated with serial half-log dilutions of ACE2 (Protein Expression Laboratory, FNLCR) as described above for CV-N binding experiments but detected with a primary rabbit anti-ACE2 monoclonal antibody (Thermo Fisher Scientific, #MA5-41038). For CV-N inhibition of ACE2-Spike WH-1 binding experiments, Spike coated wells were pre-treated with a dilution series of CV-N in PBS for 1 h, prior to washing with PBS-T and incubating with a solution of 0.2 µM ACE2.

### Western Blot Experiments.

A quantity of one microgram purified recombinant SARS-CoV-2 Spike protein (WH-1 Wuhan, BPS Bioscience #100728), S1 domain (amino acids 14 to 681, Protein Expression Laboratory, Frederick National Laboratory for Cancer Research), or S2 domain (amino acids 685-1211, Millipore Sigma #AGX820), each produced in HEK293 cells, and a quantity of 100 ng CV-N was applied to a 4 to 20% TGX SDS-PAGE gel (BioRad #4561096). After electrophoresis for 30 min at 200 volts, the SDS-PAGE gel was incubated in 20% ethanol for 5 min, and proteins were transferred to a polyvinylidene fluoride (PVDF) membrane using an iBlot 2 device (Thermo Fisher Scientific). The PVDF membrane was blocked with Intercept blocking buffer (LI-COR #927-70001), washed with PBS-T, and incubated with a 0.1 µg/mL solution of CV-N in PBS-T, 10% blocking buffer for 3 h at RT. The blot was washed three times with PBS-T and incubated with rabbit anti-CV-N antibodies for 1 h at RT. The blot was then washed with PBS-T and incubated with goat anti-rabbit IgG IRDye 680RD (LI-COR #926-68071) for 1 h at RT. After washing with PBS-T, the blot was rinsed with water and imaged using an Azure Sapphire Biomolecular Imager.

### Mapping of CV-N Oligosaccharide Binding Sites.

SARS-CoV-2 Spike S1 domain (amino acids 14 to 681, Protein Expression Laboratory, FNLCR) was combined with 100 mM Tris-HCl pH 8.0, 4 M urea, and 20 mM dithiothreitol (DTT) and heated for 1 h at 56 °C to denature the protein and reduce disulfide bonds. Iodoacetamide (IAA) was added to 50 mM, and the reaction was incubated in the dark for 1 h. Unreacted IAA was quenched by adding additional DTT to 20 mM. Reduced and alkylated S1 protein was desalted and buffer exchanged into 20 mM sodium phosphate pH 7.0, 150 mM NaCl, 0.02% (w/v) sodium azide using an Amicon ultra-15, 3 kDa ultrafiltration device (Millipore Sigma), and subsequently digested with trypsin (Millipore Sigma # 3708985001) or Glu-C (Thermo Fisher Scientific #90054) proteases for 18 h at 37 °C. Digestion reactions were heated at 95 °C for 10 min, cooled briefly to room temperature, and PMSF was added to a 1 mM final concentration. Spike S1 digests were applied to immobilized CV-N columns, prepared using C-terminally His-tagged CV-N and the His Protein Interaction Pull-Down Kit (Thermo Fisher #21277), according to manufacturer’s instructions. Eluted peptide fractions were treated with PNGase F (Thermo Fisher Scientific #A39245) at 50 °C for 1 h prior to analysis by LC-MS/MS on an Agilent 6530B Accurate Mass Q-TOF system. A quantity of 1 µg digest was applied to a PLRP-S, 2.1 × 50 mm, 5 µm, 300 Å column in 2% ACN + 0.1% formic acid, equilibrated to 40 °C at a flow rate of 0.6 mL/min. A linear gradient from 2 ACN to 60% ACN over 25 min was used to elute peptides. Peptide sequencing was accomplished using automated MS/MS acquisition software (MassHunter, Agilent) and PEAKS de novo peptide sequencing software (Bioinformatics Solutions, Inc.).

### Isothermal Titration Calorimetry.

ITC was carried out using a ITC200 device (Malvern Panalytical). For the CV-N:Spike (WH-1) experiments, 150 µM *E. coli*-produced CV-N was titrated into a calorimetry cell containing 2.5 µM trimeric Spike protein (BPS Bioscience). For the CV-N:Spike (Omicron) experiments, 150 µM CV-N was titrated into a calorimetry cell containing 3.0 µM trimeric Spike protein (Protein Expression Laboratory, FNLCR). 2.1-µL aliquots of CV-N titrant were injected into a rapidly mixing (750 rpm) solution in the calorimetry cell (volume = 200.7 µL) with a total of 19 injections during the experiment. Controls were prepared with identical amounts of titrant injected into a protein-free buffer, and control values were subtracted from the results of the other experiments. Titrations were carried out at 30 °C in 10 mM sodium phosphate buffer (pH 7.4). The isotherms, corrected for dilution/buffer effects, were fitted to a nonlinear least squares curve-fitting model (for a one-set of identical sites) using Microcal Origin v7.0 (OriginLab, Northampton, MA, USA). The extracted values for enthalpy, binding affinity and stoichiometry from the binding curve, and the free energy and entropy of interaction were calculated using Eqs. **1** and **2**:

[1] ΔG=-RTlnKa,

[2] ΔG=ΔH-TΔS,

where G is the change in Gibbs free energy, *R* is the gas constant (~1.987 cal/mol⋅K), T is the absolute temperature (303 K), *K*_a_ is the equilibrium constant, H is the change in enthalpy, and S is the change in entropy.

### Structural Modeling.

We selected the binding site residues using the ZDOCK server (51) to predict the interactions between CV-N and SARS-CoV-2 S1-RBD. The structures used for the prediction were the crystal structure of the SARS-CoV S-RBD (PDB 2GHV) and the solution NMR structure of a CV-N ensemble of 40 simulated annealing structures (PDB 2EZN). High-mannose oligosaccharides were added to SARS-CoV-2 S1-RBD, on N61, N122 and N234, using GLYCAM-Web. Protein interactions were visualized using DS Visualizer (Biovia).

### Syrian Golden Hamster Model of SARS-CoV-2 Infections.

Syrian golden hamster studies were performed by BioQual (Boulder, CO) under contract. All animal experiments were approved by the BIOQUAL Inc. Institutional Animal Care and Use Committee and performed in an AAALAC-approved facility

CV-N was diluted in MEM to a stock concentration of 5 mg/mL and delivered intranasally to lightly anesthetized animals. In this work, we conducted two distinct CV-N treatment protocols. In the pilot study (*SI Appendix*, Table S3), 24 animals, six individuals per group (three males/three females), were intranasally challenged with SARS-CoV-2 on Study Day (SD) 0 and treated with CV-N: Groups 2 to 5 with minimal essential medium (MEM) and Group 5 intranasally twice daily on Study Days 0 to 4. CV-N was administered at 1.0 mg/kg, 0.2 mg/kg, and 0.04 mg/kg twice daily pre-SARS-CoV-2 challenge in Groups 2, 3, and 4, respectively. In Groups 1 and 5, MEM only was administered on Study Days 0 to 4. Group 1 was not challenged with SARS-CoV-2. The animals were weighed every day for 7 d to monitor weight loss. After this period, animals were euthanized and lung tissue was collected for histopathology and viral RNA detection by RT-PCR.

The second study design, referred to as “one-shot” protocol, was carried out as follows. Briefly, a total of 24 golden Syrian hamsters were evaluated in the study. In Group 1, twelve animals (six males/six females) were MEM-treated controls (Sham) with SARS-CoV-2 intranasal challenge on SD 0. In Group 2, twelve animals (six males/six females) were treated with a single CV-N dose of 2 mg/kg intranasally on Day 0 prior to i.n. SARS-CoV-2 challenge. Six animals from each group were euthanized for lung tissue collection on Day 4, and the other six were euthanized on Day 7. Histopathology and RT-PCR was performed on twelve animals; twelve animals were RT-PCR only. Specific details for the TCID-50 assay, viral RNA extraction and quantification using qRT-PCR, and subgenomic mRNA assay are found in the *SI Appendix*.

### Histopathology.

Histopathological evaluation of lung sections was performed by board-certified pathologists at Experimental Pathology Laboratories, Inc. (Sterling, VA) for SARS-CoV-2-related findings. At necropsy, organs were collected and placed in 10% neutral buffered formalin for histopathologic analysis. Tissues were processed through to paraffin blocks, sectioned once at ~5 μm thickness, and stained with hematoxylin/eosin.

## Supplementary Material

Appendix 01 (PDF)

## Data Availability

All study data are included in the article and/or *SI Appendix*.
