# Supplementary material for: Cyanovirin-N binds to select SARS-CoV-2 spike oligosaccharides outside of the receptor binding domain and blocks infection by SARS-CoV-2
Source: Proc Natl Acad Sci U S A. 2023 Feb 28;120(10):e2214561120. doi: 10.1073/pnas.2214561120 (PMC10013841; doi:10.1073/pnas.2214561120)

SUPPLEMENTARY MATERIALS

Item **Page**

1. **Detailed Materials and Methods** **1**

2. **References** **12**

3. **Supplementary Figure 1 - Initial data showing inhibition of SARS-CoV-2 (Wuhan) pseudovirus infection by cyanovirin-N.** **13**

4. **Supplementary Table 1 - Information on tested SARS-CoV-2 strains.** **14**

5. **Supplementary Figure 2 - Cyanovirin-N (CV-N) shows activity against various replicating SARS-CoV-2 strains by plaque reduction assay.** **15**

6. **Supplementary Table 2 - ITC results of CV-N binding to baculovirus and HEK293 cell produced SARS-CoV-2 Spike** **16**

7. **Supplementary Table 3 - Syrian golden hamster experimental design.** **16**

8. **Supplementary Figure 3 - Clinical and virologic evaluation of the pilot experiment for CV-N dosing on Syrian golden hamster following SARS-CoV-2 i.n. challenge.** **17**

9. **Supplementary Table 4 - Syrian golden hamster CV-N "*one-shot*" treatment experimental design.** **18**

10. **Supplementary Figure 4. A single CV-N Intranasal administration protects both (A) female and (B) male Syrian golden hamsters from weight loss following SARS-CoV-2 challenge.** **18**

1. Detailed Materials and Methods

Sources of pure lectins and viral proteins

The recombinant purified lectins produced in bacteria were provided by the National Cancer Institute. The SARS-CoV-2 S1 protein produced in baculovirus-infected insect cells was sourced from Sino Biological (catalog no. 40150-V08B1). His-tagged HEK293-produced trimeric Spike (S1+S2) was purchased from BPS Bioscience catalog # 100728. HEK293-produced SARS-CoV-2 Spike glycoprotein isolated domains S1 and S2 were either produced by the Protein Expression Laboratory, Frederick National Laboratory for Cancer Research (S1) or purchased from Millipore Sigma catalog # AGX820 (S2). CV-N was produced in the Molecular Targets Program, Center for Cancer Research, NCI.

SARS-CoV and SARS-CoV-2 pseudovirus-neutralization assay (TYPE 1)

HEK-293T cells (ATCC repository) were maintained in Dulbecco's modified Eagle's medium (DMEM) with 10% fetal bovine serum, 100 IU/mL penicillin and 100 µg/mL streptomycin (Thermo Fisher Scientific). HEK-293T cells overexpressing human ACE2 were a gift from Integral Molecular Company and maintained in DMEM as above, also containing 1 µg/mL puromycin (Thermo Fisher Scientific). Pseudoviruses were prepared as previously reported (*1*). Briefly, HIV-1 luciferase reporter pseudoviruses expressing SARS-CoV-2 S protein were generated using plasmids pNL4-3.Luc.R-.E- (NIH AIDS repository) and SARS-CoV-2.SctΔ19 prepared by GeneArt (Thermo Fisher Scientific) from a human-codon optimized SARS-CoV-2 S-protein sequence from the indicated VOCs minus the C-terminal 19 amino acids inserted into vector pcDNA3.4-TOPO. HEK-293T cells were transfected with SARS-CoV-2.SctΔ19 using X-tremeGENE HP (Merck, Darmstadt, Germany), and 24 h later they were super-transfected with pNL4-3.Luc.R-.E-. Supernatants were harvested after 48 h, passed through 0.45-µM filters (Merck) and stored at –80 °C. The p24gag content of all viruses was quantified by ELISA (Perkin Elmer) and viruses were titrated in HEK-293T cells overexpressing ACE2.

HEK-293T cells overexpressing ACE2 were used to test the antiviral effect of lectins at the indicated concentrations against SARS-CoV-2 pseudoviruses in duplicate. The inhibitory capacity of the lectins was assessed after 48 hours using the EnSight Multimode Plate Reader and BriteLite Plus Luciferase reagent (PerkinElmer, USA). The values were normalized, and the ID_{50} (reciprocal dilution inhibiting 50% of the infection) was calculated by plotting and fitting all duplicate neutralization values and the log of plasma dilution to a 4-parameters equation in Prism 9.0.2 (GraphPad Software, USA) as previously described (*2*).

SARS-CoV-2 pseudovirus-neutralization assay (TYPE 2)

The second neutralization assay was based on the VSV pseudoviral system (Kerafast). We seeded 3×10^6 HEK-293T cells in 100-mm wells 24 h before transfection with 20 µg of vector pGBW-m4137382 (Addgene #149539, kindly provided by Ginkgo Bioworks) containing the SARS-CoV-2 S-protein sequence minus the C-terminal 19 amino acids, and 60 µl of Fugene HD (Promega) in 1 mL Opti-MEM medium (Thermo Fisher Scientific) at room temperature for 15 min. After 48 h at 37 °C in a 5% CO_2 atmosphere, the medium was removed and replaced with 8 ml medium containing VSV G*ΔG-luciferase from the Kerafast system at a multiplicity of infection (MOI) of 3. The cells were incubated for 2 h as above then washed three times with warm PBS and incubated with 10 ml of fresh medium for 24 h as above. Finally, pseudoviruses were collected from the medium, centrifuged at 3000 × g for 5 min, passed through a 0.45-µm filter and stored at –80 °C.
Vero E6 cells were seeded into 96-well plates (7500 cells per well) and incubated for 24 h. Neutralization was tested by mixing 100 µl/well of viruses (see above) and 100 µl/well of each lectin at different

concentrations diluted in PBS and incubated for 1 h at 37 °C. Before pseudoviral transduction, Vero E6 cells were washed twice with warm PBS. Lectin-pseudovirus mixtures were added to the cells and incubated for 72 h at 37 °C in a 5% CO_2 atmosphere. After 72 h, cells were incubated with PrestoBlue HS Reagent (Thermo Fisher Scientific) in PBS at 37 °C for 30 min to determine cell viability. Luciferase substrate (Promega) added to determine infectivity. Fluorescence and luminescence were detected using an Infinite M200 plate reader (Tecan). Infectivity was normalized by cell viability and expressed as a percentage relative to the control (PBS only, without lectins) in triplicate.

Cyanovirin-N cytotoxicity on Vero cells

To determine the tolerable CV-N concentrations, two treatment protocols were performed on Vero cells. Previously, cells were seeded in 96-well plates at 1 x 10^4 cells/well density. In the first protocol, serial 2.5-fold dilutions of CV-N (from 1.14 x 10^4 to 7.44 nM, i.e. 125 to ~0.08 µg/mL) were maintained in culture medium (DMEM supplemented with 1.5 % FBS) for 72 h. In order to emulate the plaque reduction treatment conditions, the second protocol consisted of incubating CV-N dilutions with Vero cells for 1 h in the incubator (37 °C; 5 % CO_2) in low medium volumes (50 µL of DMEM without FBS per well). After this period, CV-N was removed and cells were grown in fresh DMEM supplemented with 1.5 % FBS (100 µL/well) for 72h. In both protocols, CellTiter Blue reagent (Promega) was added in each well, incubated for 3 h, and fluorescence was measured (560/590 nm) using GloMax® Discover Microplate Reader (Promega). Data obtained from four independent experiments were plotted as Mean ± SEM and analyzed by Two-Way ANOVA test using GraphPad Prism 8 software. Relative viability curves of the two protocols were compared by Sydak post-hoc test.

Plaque reduction assay against replicating SARS-CoV-2 strains

Experiments handling replicative competent viruses were performed in a Biosafety Level 3 (BSL-3) laboratory located in the Laboratório de Virologia Molecular (UFRJ), Brazil. To address CV-N inhibitory effect on SARS-CoV-2, we compared the sensitivity to CV-N of nine SARS-CoV-2 isolates representative of previously or currently circulating variants by plaque reduction assay. Information about these SARS-CoV-2 established strains are summarized in Supplementary Table 1. The assay was conducted similarly to the PRNT assay commonly used to assess antibody neutralization titers against a virus. Experiment readout was given by plaque forming units (PFU) count and normalization to the untreated control inoculum.

Briefly, serial 5-fold dilutions of CV-N (from 4.65 x 10^{-3} to 1.82 x 10^3 nM, i.e., 20 to 5.12 x 10^{-5} µg/mL) were incubated with 50-200 PFUs of SARS-CoV-2 suspension for 1 hour at 37 °C in the incubator. After incubation, CV-N:SARS-CoV-2 suspensions were added to Vero cells (ATCC CCL-81) confluent monolayers seeded in 12-well plates to allow viral adsorption for 1 hour at 37 °C in the incubator. After the adsorption step, inoculum was removed, and cells were grown in semisolid Alpha-MEM (1.25 % carboxymethylcellulose; 1.5 % fetal bovine serum; 1 % Pen-Strep in Alpha-MEM) for three to four days in the incubator, depending on viral strain. After infection, 10 % formaldehyde solution was added onto semisolid medium and cells were fixed for 1 hour at room temperature. Fixed Vero cells monolayers were then washed and stained with crystal violet solution (0.5 % w/v crystal violet; 20 % v/v ethanol) for 15 minutes to allow PFU visualization and quantitation. Normalized results were expressed by Mean ± SEM and analyzed by non-linear regression (best-fit method) using GraphPad Prism 8 to calculate IC_{50} values. IC_{50} and Hill-slope values obtained were imputed to estimate IC_{90} values for each SARS-CoV-2 isolate tested using Graphpad QuickCalcs - accessed in April 2022.

Omicron adsorption assay

To evaluate the impact of CV-N treatment on SARS-CoV-2 adsorption and entry, replicating isolate OmiSP (Omicron - BA.1 variant isolated in Brazil) was pre-incubated with 0.1-100 nM final concentration of CV-N at 37 °C for 1 hour, including a mock-treated (DMEM) control. Viral suspension was diluted to obtain a final inoculum at a multiplicity of infection (MOI) of 0.1. After incubation, the CV-N/OmiSP suspension was refrigerated until 4 °C. Vero E6 cells expressing hACE-2 / hTMPRSS2 (Vero E6-TMPRSS2-T2A-ACE2; NR-54970) were seeded in 24 wells plate (Corning) at a density of 10^5 cells per well, provided with DMEM High glucose (GIBCO) supplemented with 5% FBS. Cell monolayers were pre-chilled at 4 °C prior to inoculation with CV-N/OmiSP suspension. To inoculate the Vero E6 monolayers, conditioned medium was removed and 120 µL of CV-N/OmiSP suspension was added and plates were incubated at 4°C for 1 hour, being gently rocked every 15 minutes. After the adsorption step, cells were washed three times with ice-cold PBS to remove unadsorbed viral particles. For the time-point of 0 hour post adsorption, cells were directly lysed for RNA extraction after PBS washes. For the later time-points (0.5, 6 and 12 hours post adsorption), culture medium was provided and cells were incubated at 37 °C. At collection time, conditioned medium was discarded and cells monolayers were washed with PBS prior to RNA extraction. For RNA extraction, cells were processed with ReliaPrep™ Viral TNA Miniprep System (Promega), following manufacturer's procedures.

SARS-CoV-2 genomic RNA count

To measure gRNA copies in Vero E6 cells infected with SARS-CoV-2 (OmiSP - Omicron variant) lysate, RT-qPCR was performed using GoTaq Probe 1-Step RT-qPCR System (Promega; cat. A612B), using primers and probes to amplify a conserved region of the N gene of SARS-CoV-2. RNA copies quantification was performed by interpolating obtained Ct values in a standard curve of the synthetic N gene amplified template (Integrated DNA Technologies) with RNA copies ranging from 2-20,000 copies per µL.

Primers/probe sequences:

2019-nCoV_N1-F :5'-GAC CCC AAA ATC AGC GAA AT-3'

2019-nCoV_N1-R: 5'-TCT GGT TAC TGC CAG TTG AAT CTG-3'

2019-nCoV_N1-P: 5'-FAM-ACC CCG CAT TAC GTT TGG TGG ACC-BHQ1-3'

SARS-CoV-2 subgenomic RNA relative expression

To evaluate SARS-CoV-2 transcription levels after CV-N treatment, Vero E6 cells inoculated with OmiSP isolate were lysed for total RNA extraction. cDNA was synthesized using High-Capacity cDNA Reverse Transcription Kit (Thremo, cat. 4368814), following manufacturer's cycle parameters. Specific primers and probes that differentiate N gene subgenomic transcripts were applied. As an endogenous control, GAPDH gene was amplified with SYBR™ Green PCR Master Mix using specific primers for mature mRNA transcript. After amplification, obtained Ct values were used to calculate fold-change by $2^{-\Delta\Delta Ct}$ method. Each time-point curve was normalized by its respective untreated control, meaning that all samples collected at a determined time-point were normalized to the untreated infected control respective of that time-point.

Primers/probe sequences:

N_sgRNA-F: 5'-CGA TCT CTT GTA GAT CTG TTC TCT AAA CGA ACT TAT GTA CTC-3'

N_sgRNA-R: 5'-ATA TTG CAG CAG TAC GCA CAC A-3'

N_sgRNA-Probe: 5'-ACA CTA GCC ATC CTT ACT GCG CTT CG-3'

GAPDH-F: 5'-GTG GAC CTG ACC TGC CGT CT-3'

GAPDH-R: 5'-GGA GGA GTG GGT GTC GCT GT-3'

Genome assembly, annotation and phylogenetic analysis

Sequencing reads were processed using a previously described pipeline (*3*). Consensus genome sequences were then annotated with NextClade (*4*) and lineage classification was conducted with Pangolin (*5*). To further contextualize the diversity of viral isolates used in our experiments, we conducted a phylogenetic analysis with a comprehensive and representative set of SARS-CoV-2 genome sequences available on GISAID EpiCoV database (n = 401). This reference dataset was assembled by gathering the 50 sequences closest to each of ours removing repeated sequences (Audacity instant application, as of 19 April 2022). Sequences were aligned using MAFFT v v7.475 (6) and a maximum likelihood tree was inferred with IQ-Tree 2 (*7*) under the GTR+F+I+G4 model (*8*).

Calculation of IC_{50} for CV-N using different SARS-CoV-2 VOC on Vero E6 cells (Spain)

Biosafety Approval**.** The biologic biosafety committee of the Research Institute Germans Trias i Pujol approved the execution of SARS-CoV-2 experiments at the BSL3 laboratory of the Center for Bioimaging and Comparative Medicine (CSB-20-015-M3).

Cell culture and viral isolation and titration.

Vero E6 cells (ATCC CRL-1586) were cultured in Dulbecco's modified Eagle medium (Invitrogen) supplemented with 10% fetal bovine serum (FBS; Invitrogen), 100 U/ml penicillin, 100 µg/ml streptomycin, (all from Invitrogen). SARS-CoV-2 were isolated from a nasopharyngeal swab collected in March 2020 in Spain in Vero E6 cells as described (*9*). VOCs were propagated for two passages and a virus stock was prepared collecting the supernatant from Vero E6 and sequenced. Genomic sequences were deposited at GISAID repository (http:// gisaid.org) with the following accession numbers: B.1 (D614G) isolated in Spain in March 2020 (EPI_ISL_510689); 2 VOC isolated in Spain from January to February 2021: Alpha or B.1.1.7 (EPI_ISL_1663569) and Delta or B.1.617.2 (originally detected in India; EPI_ISL_3342900), and Omicron or B.1.1.529 isolated in Spain in December 2021 (originally detected in South Africa; EPI_ISL_8151031). Genomic sequencing was performed from viral supernatant by using standard ARTIC v3 or v4 based protocols followed by Illumina sequencing (dx.doi.org/10.17504/protocols.io.bhjgj4jw). Raw data analysis was performed by viralrecon pipeline (https://github.com/nf-core/viralrecon) while consensus sequence was called using samtools/ivar at the 75% frequency threshold. Viral stocks were titrated in 3-fold serial dilutions on Vero E6 cells to achieve a 50% of viral-induced cytopathic effect 3 days post-inoculation with all VOC estimated. The viral-induced cytopathic effect was measured using CellTiter-Glo Luciferase reagent (Promega) and a Luminoskan Plate Reader from Thermofisher.

Determination of antiviral activity.

To determine the IC_{50}, the indicated serial dilutions of CV-N were added to 60,000 Vero E6 cells per well in 96 well plates in duplicates to determine the viral-induced cytopathic effect 72h later. The relative light units (RLU) were normalized, and the IC_{50} (the reciprocal concentration inhibiting 50% of the infection) was calculated by plotting and fitting the log of CV-N concentration vs. cytotoxicity to a 4-parameter equation in Prism 9. In parallel, cells exposed to CV-N in the absence of virus were assayed to detect any possible drug-induced cytotoxic effect using CellTiter-Glo Luciferase reagent.

SARS-CoV-2 adsorption assay

To evaluate the impact of CV-N treatment on SCoV-2 adsorption and entry, replicating isolate OmiSP (Omicron - BA.1 variant isolated in Brazil) was pre-incubated with 0.1-100 nM final concentration of CV-

N at 37 °C for 1 hour, including a mock-treated (DMEM) control. Viral suspension was diluted to obtain a final inoculum at a multiplicity of infection (MOI) of 0.1. After incubation, the CV-N/OmiSP suspension was refrigerated until 4 °C. Vero E6 cells expressing hACE-2 / hTMPRSS2 (Vero E6-TMPRSS2-T2A-ACE2; NR-54970) were seeded in 24 wells plate (Corning) at a density of 10^5 cells per well, provided with DMEM High glucose (GIBCO) supplemented with 5% FBS. Cell monolayers were pre-chilled at 4 °C prior to inoculation with CV-N/OmiSP suspension. To inoculate the Vero E6 monolayers, conditioned medium was removed and 120 µL of CV-N/OmiSP suspension was added and plates were incubated at 4°C for 1 hour, being gently rocked every 15 minutes. After the adsorption step, cells were washed three times with ice-cold PBS to remove unadsorbed viral particles. For the time-point of 0 hour post adsorption, cells were directly lysed for RNA extraction after PBS washes. For the later time-points (0.5, 6 and 12 hours post adsorption), culture medium was provided and cells were incubated at 37 °C. At collection time, conditioned medium was discarded and cells monolayers were washed with PBS prior to RNA extraction. For RNA extraction, cells were processed with ReliaPrep™ Viral TNA Miniprep System (Promega), following manufacturer’s procedures.

SARS-CoV-2 genomic RNA count

To measure gRNA copies in Vero E6 cells infected with SARS-CoV-2 (OmiSP - Omicron variant) lysate, RT-qPCR was performed using GoTaq Probe 1-Step RT-qPCR System (Promega; cat. A612B), using primers and probes to amplify a conserved region of the N gene of SARS-CoV-2. RNA copies quantification was performed by interpolating obtained Ct values in a standard curve of the synthetic N gene amplified template (Integrated DNA Technologies) with RNA copies ranging from 2-20,000 copies per µL.

Primers/probe sequences:

2019-nCoV_N1-F :5’-GAC CCC AAA ATC AGC GAA AT-3’

2019-nCoV_N1-R: 5’-TCT GGT TAC TGC CAG TTG AAT CTG-3’

2019-nCoV_N1-P: 5’-FAM-ACC CCG CAT TAC GTT TGG TGG ACC-BHQ1-3’

SARS-CoV-2 subgenomic RNA relative expression

To evaluate SARS-CoV-2 transcription levels after CV-N treatment, Vero E6 cells inoculated with OmiSP isolate were lysed for total RNA extraction. cDNA was synthesized using High-Capacity cDNA Reverse Transcription Kit (Thremo, cat. 4368814), following manufacturer's cycle parameters. Specific primers and probes that differentiate N gene subgenomic transcripts were applied. As an endogenous control, GAPDH gene was amplified with SYBR™ Green PCR Master Mix using specific primers for mature mRNA transcript. After amplification, obtained Ct values were used to calculate fold-change by $2^{-\Delta\Delta Ct}$ method. Each time-point curve was normalized by its respective untreated control, meaning that all samples collected at a determined time-point were normalized to the untreated infected control respective of that time-point.

Primers/probe sequences:

N_sgRNA-F: 5’-CGA TCT CTT GTA GAT CTG TTC TCT AAA CGA ACT TAT GTA CTC-3’

N_sgRNA-R: 5’-ATA TTG CAG CAG TAC GCA CAC A-3’

N_sgRNA-Probe: 5’-ACA CTA GCC ATC CTT ACT GCG CTT CG-3’

GAPDH-F: 5’-GTG GAC CTG ACC TGC CGT CT-3’

GAPDH-R: 5’-GGA GGA GTG GGT GTC GCT GT-3’

<u>*ELISA Assays*</u>

For ELISA assays, purified, recombinant SARS-CoV-2 Spike protein (S1+S2 trimer, Wuhan, BPS Bioscience #100728) or Spike receptor binding domain (Wuhan, BEI Resources #NR-52306), both produced in HEK293 cells, were immobilized on high-binding ELISA plates (Greiner #655081). Plates were washed with 1X PBS pH 7.4, 0.05% Tween-20 (PBS-T) and blocked with a solution of 5% (w/v) bovine serum albumin (BSA, Fisher #BP9706-100) in 1X PBS pH 7.4 (PBS). For evaluating the binding of CV-N to Spike and RBD, plates were washed three time with PBS-T and incubated with serial half-log dilutions of CV-N, diluted in PBS, for one hour at room temperature. Plates were washed three times with PBS-T and incubated with rabbit anti-CV-N polyclonal antibodies (*10*) for one hour at room temperature. Plates were then washed three times with PBS-T and incubated with goat anti-rabbit IgG-HRP conjugate (Thermo Fisher Scientific #31460) for one hour at room temperature. Plates were washed three times with PBS-T and developed using 1-Step Ultra TMB-ELISA solution (Thermo Fisher Scientific #34028). The HRP reaction was stopped with 1M hydrochloric acid and absorbance values at 450 nm were measured on a SpectraMax i3x plate reader (Molecular Devices).

For evaluating ACE2 binding to Spike, after Spike immobilization and BSA blocking, plates (Greiner #781061) were washed three times and incubated with a half-log dilution series of ACE2 (Protein Expression Laboratory, Frederick National Laboratory for Cancer Research) produced in HEK293 cells. Plates were incubated for one hour at room temperature and washed with PBS-T. Binding of ACE2 was detected using rabbit anti-human ACE2 monoclonal antibodies (Thermo Fisher Scientific #MA5-41038) Plates were washed, incubated with goat anti-rabbit IgG HRP conjugate, and developed as described above. For evaluating the effect of CV-N on ACE2 binding to Spike, wells were incubated with a half-log dilution series of CV-N, diluted in PBS, for one hour at room temperature prior to the addition of ACE2 (5 pmol/well). ACE2 binding was detected as described above.

Western blot experiments

A quantity of one microgram purified recombinant SARS-CoV-2 Spike protein (S1+S2 trimer, Wuhan, BPS Bioscience #100728), S1 domain (amino acids 14-681, Protein Expression Laboratory, Frederick National Laboratory for Cancer Research), or S2 domain (amino acids 685-1211, Millipore Sigma #AGX820), each produced in HEK293 cells, and a quantity of 100 ng CV-N was applied to a 4-20% TGX SDS-PAGE gel (BioRad #4561096). After electrophoresis for 30 minutes at 200 volts, the SDS-PAGE gel was incubated in 20% ethanol for five minutes, and proteins were transferred to a PVDF membrane using an iBlot 2 device (Thermo Fisher Scientific). The PVDF membrane was blocked with Intercept (PBS) blocking buffer (LI-COR #927-70001), washed with PBS-T, and incubated with a 0.1 μg/ml solution of CV-N in PBS-T, 10% blocking buffer for three hours at room temperature. The blot was washed three times with PBS-T and incubated with rabbit anti-CV-N antibodies (see ELISA section) for one hour at room temperature. The blot was then washed with PBS-T and incubated with goat anti-rabbit IgG IRDye 680RD (LI-COR #926-68071) for one hour at room temperature. After washing with PBS-T, the blot was rinsed with water and imaged using an Azure Sapphire Biomolecular Imager.

Mapping of CV-N oligosaccharide binding sites

SARS-CoV-2 Spike S1 domain (amino acids 14-681, Protein Expression Laboratory, Frederick National Laboratory for Cancer Research) was combined with 100 mM Tris-HCl pH 8.0, 4M urea, and 20 mM DTT and heated for one hour at 56 °C to denature the protein and reduce disulfide bonds. Iodoacetamide (IAA) was added to 50 mM and the reaction was incubated in the dark for one hour. Unreacted IAA was quenched by adding additional DTT to 20 mM. Reduced and alkylated S1 protein was desalted and buffer exchanged into 20 mM sodium phosphate pH 7.0, 150 mM NaCl, 0.02% (w/v) sodium azide using an

Amicon ultra-15, 3 kDa ultrafiltration device (Millipore Sigma), and subsequently digested with trypsin (Millipore Sigma # 3708985001) or Glu-C (Thermo Fisher Scientific #90054) proteases for 18 hours at 37 °C. Digestion reactions were heated at 95 °C for ten minutes, cooled briefly to room temperature and PMSF was added to a 1 mM final concentration. Spike S1 digests were applied to immobilized CV-N columns, prepared using c-terminally his-tagged CV-N (*11*) and the His Protein Interaction Pull-Down Kit (Thermo Fisher #21277), according to manufacturer's instructions. Eluted peptide fractions were treated with PNGase F (Thermo Fisher Scientific #A39245) at 50 °C for one hour prior to analysis by LC-MS/MS on an Agilent 6530B Accurate Mass Q-TOF system. A quantity of 1 µg digest was applied to a PLRP-S, 2.1 x 50 mm, 5 µm, 300Å column in 2% ACN + 0.1% formic acid, equilibrated to 40 °C at a flow rate of 0.6 ml/min. A linear gradient from 2% ACN to 60% ACN over 25 minutes was used to elute peptides. Peptide sequencing was accomplished using automated MS/MS acquisition software (MassHunter, Agilent) and PEAKS *de novo* peptide sequencing software (Bioinformatics Solutions, Inc.).

Isothermal titration calorimetry

ITC was carried out using a ITC200 device (Malvern Panalytical, Malvern, UK). For the CVN:Spike (Wuhan) titration experiments, 150 µM *E. coli*-produced CV-N was titrated into a calorimetry cell containing 2.5 µM HEK293-produced trimeric Spike protein (BPS Bioscience). For the CV-N:Spike (Omicron) titration experiments, 150 µM *E. coli*-produced CV-N was titrated into a calorimetry cell containing 3.0 µM HEK293-produced trimeric Spike protein (Protein Expression Laboratory, FNLCR). In a typical experiment, 2.1-µL aliquots of CV-N titrant were injected into a rapidly mixing (750 rpm) solution in the calorimetry cell (volume = 200.7 mL) with a total of 19 injections during the experiment. Controls were prepared with identical amounts of titrant injected into a protein-free buffer, and control values were subtracted from the results of the other experiments. Titrations were carried out at 30 °C in 10 mM sodium phosphate buffer (pH 7.4). The isotherms, corrected for dilution/buffer effects, were fitted to a nonlinear least squares curve-fitting model (for a 1-set of identical sites) using Microcal Origin v7.0 (OriginLab, Northampton, MA, USA). The extracted values for enthalpy, binding affinity and stoichiometry from the binding curve, and the free energy and entropy of interaction were calculated using Equations (1) and (2):

$$\Delta G = -RT\ln K_a \qquad (1)$$

$$\Delta G = \Delta H - T\Delta S \qquad (2)$$

where ΔG is the change in Gibbs free energy, *R* is the gas constant (~1.987 cal/mol·K), T is the absolute temperature (303 K), K_a is the equilibrium constant, ΔH is the change in enthalpy and ΔS is the change in entropy.

Structural modeling

We selected the binding site residues using the ZDOCK server (*12*) to predict the interactions between CV-N and SARS-CoV-2 S1-RBD. The structures used for the prediction were the crystal structure of the SARS-CoV S-RBD (PDB 2GHV) and the solution NMR structure of a CV-N ensemble of 40 simulated annealing structures (PDB 2EZN). High-mannose oligosaccharide were added to SARS-CoV-2 S1-RBD, on N61, N122 and N234, using GLYCAM-Web. Protein interactions were visualized using DS Visualizer (Biovia).

Syrian golden hamster model of SARS-CoV-2 infections

Syrian golden hamster studies were performed by BioQual (Boulder, CO) under contract. All animal experiments were approved by the BIOQUAL Inc. Institutional Animal Care and Use Committee and performed in an AAALAC-approved facility.

To investigate the ability of CV-N to protect Syrian hamsters from an in vivo challenge, the CV-N protein was diluted in MEM to a stock concentration of 5 mg/mL and delivered intranasally to lightly anesthetized animals. In this work, we conducted two distinct CV-N treatment protocols. In the pilot study (**Supplementary Table 3**), 24 animals, six individuals per group (3 males/3 females), were intranasally challenged with SARS-CoV-2 on Study Day (SD) 0 and treated with CV-N: Groups 2-5 with minimal essential medium (MEM); and Group 5 intranasally twice daily on Study Days 0-4. CV-N was administered at 1.0 mg/kg, 0.2 mg/kg, and 0.04 mg/kg twice daily pre-SARS-CoV-2 challenge in Groups 2, 3 and 4, respectively. In Groups 1 and 5, MEM only was administered on Study Days 0-4. Group 1 was not challenged with SARS-CoV-2. The animals were weighed every day for 7 days to monitor weight loss. After this period, animals were euthanized and lung tissue was collected for histopathology and viral RNA detection by RT-PCR.

The second study design, referred to as "one-shot" protocol, was carried out as follows (**Supplementary Table 4**). Briefly, a total of 24 Golden Syrian hamsters were evaluated in the study. In Group 1, twelve animals (6 males/6 females) were MEM-treated controls (Sham) with SARS-CoV-2 intranasal challenge on SD 0. In Group 2, twelve animals (6 males/ 6 females), were treated with a single CV-N dose of 2 mg/kg intranasally on Day 0 prior to i.n. SARS-CoV-2 challenge. Six animals from each group were euthanized for lung tissue collection on Day 4 and the other six were euthanized on Day 7. Histopathology and RT-PCR was performed on twelve animals; twelve animals were RT-PCR only.

TCID50 Assay

For infectious titer determination from tissue samples, TCID50 assay was performed. To set up the assay, frozen lung tissue was placed in 15 mL conical tube on wet ice containing 0.5 mL media and homogenized 10-30 secs (Probe, Omni International: 32750H). The tissue homogenate was spun to remove debris at 2000g ,4°C for 10 min. The supernatant was passed through a strainer that is placed on original vial, placing vials on wet ice. 20 µL of this supernatant was tested in the assay in quadruplicate in a 96 well plate format.

To perform this assay, Vero TMPRSS2 cells were plated at 25,000 cells per well in DMEM + 10% FBS + Gentamicin. The plate was incubated at 37°C, 5.0% CO_2. The cells were 80-100% confluent the following day. When the 80-100% is confirmed, the media was aspirated out and replaced with 180µL of DMEM + 2% FBS + gentamicin. 20 µL of the sample was added to top row in quadruplicate. The top row was mixed 5 times with a pipette and titered down 20 µL, representing 10-fold dilutions. The pipette tips are disposed of between each row and the mixing is repeated until the last row on the plate. The plates for the samples were incubated again at 37°C, 5.0% CO_2 for 4 days. After 4 days, the plates are visually inspected for CPE. Non-infected wells would have a clear confluent cell layer. Infected cells would have cell rounding. The presence of CPE was recorded as a plus (+) and absence of CPE as minus (-). The $TCID_{50}$ is then calculated using the Read-Muench formula. For optimal assay performance, the $TCID_{50}$ of the positive control should test within 2-fold of the expected value.

Viral RNA extraction and quantification using qRT-PCR

Genomic mRNA PCR Assay

The qRT-PCR assay utilizes primers and a probe specifically designed to amplify and bind to a conserved region of Nucleocapsid gene of Coronavirus. The signal is compared to a known standard curve and calculated to give copies per mL. For the qRT-PCR assay, viral RNA is first isolated from an oral swab

using the Qiagen MinElute virus spin kit (cat. no. 57704). For tissues it is extracted with RNA-STAT 60 (Tel-test"B")/ chloroform, precipitated and resuspended in RNAse-free water. To generate a control for the amplification reaction, RNA is isolated from the applicable SARs-CoV-2 stock using the same procedure. The amount of RNA was determined from an O.D. reading at 260, using the estimate that 1.0 OD at A260 equals 40 μg/mL of RNA. With the number of bases known and the average base of RNA weighing 340.5 g/mole, the number of copies was then calculated, and the control diluted accordingly. A final dilution of 10^8 copies per 3 μL was then divided into single use aliquots of 10 μL. These were stored at -80ºC until needed. Several aliquots were chosen at random and compared to previous controls to verify consistency. For the master mix preparation, 2.5 mL of 2X buffer containing Taq-polymerase, obtained from the TaqMan RT-PCR kit (Bioline cat# BIO-78005), was added to a 15 mL tube. From the kit, 50 μL of the RT and 100 μL of RNAse inhibitor was also added. The primer pair at 2 μM concentration was then added in a volume of 1.5 mL. Lastly, 0.5 mL of water and 350 μL of the probe at a concentration of 2 μM were added and the tube vortexed. For the reactions, 45 μL of the master mix and 5 μL of the sample RNA was added to the wells of a 96-well plate. All samples were tested in triplicate. The plates were sealed with a plastic sheet.

For control curve preparation, samples of the control RNA were obtained from the –80ºC freezer. The control RNA was prepared to contain 10^6 to 10^7 copies per 3 μL. Eight 10-fold serial dilutions of control RNA is prepared using RNAse-free water by adding 5 μL of the control to 45 μL of water and repeating this for 7 dilutions. This gives a standard curve with a range of 1 to 10^7 copies/reaction. Duplicate samples of each dilution were prepared as described above. If the copy number exceeded the upper detection limit, the sample was diluted as needed. For amplification, the plate was placed in an Applied Biosystems 7500 Sequence detector and amplified using the following program: 48ºC for 30 minutes, 95ºC for 10 minutes followed by 40 cycles of 95ºC for 15 seconds, and 1 minute at 55ºC. The number of copies of RNA per mL was calculated by extrapolation from the standard curve and multiplied by the reciprocal of 0.2 mL extraction volume. This gave a practical range of 50 to 5 x 10^8 RNA copies per gram tissue.
Primers/probe sequences:
2019-nCoV_N1-F :5'-GAC CCC AAA ATC AGC GAA AT-3'
2019-nCoV_N1-R: 5'-TCT GGT TAC TGC CAG TTG AAT CTG-3'
2019-nCoV_N1-P: 5'-FAM-ACC CCG CAT TAC GTT TGG TGG ACC-BHQ1-3'

Sub-genomic mRNA Assay
For the qRT-PCR assay, hamster lung tissue is homogenized in BioSpec 2ml bead beater tubes (Cat# 10831-V) using 1mL TrIzol followed by phase separation (100μL Bromochloropropane), precipitated and resuspended in AVE Buffer (Qiagen 1020953). The qRT-PCR assay utilizes primers and a probe specifically designed to amplify and bind to a conserved region of Nucleocapsid (N) gene of Coronavirus. The signal is compared to a known standard curve and calculated to give copies per mL. To generate a control for the amplification reaction, RNA is isolated from the applicable virus stock using the Qiagen MinElute virus spin kit (cat. no. 57704). The amount of viral RNA is determined comparing it to a known quantity of Plasmid control. A final dilution of 10^8 copies per 3 μL is then divided into single use aliquots of 10 μL and stored at -80°C. For the master mix preparation, 2.5 ml of 2X buffer containing Taq-polymerase, obtained from the TaqMan RT-PCR kit (Bioline #BIO-78005), is added to a 15 ml tube. From the kit, 50 µl of the RT and 100 µl of RNAse inhibitor is also added. The primer pair at 2 μM concentration is then added in a volume of 1.5 ml. Lastly, 0.5 ml of water and 350 µl of the probe at a concentration of 2 μM are added and the tube vortexed. For the reactions, 45 µl of the master mix and 5 µl of the sample

RNA are added to the wells of a 96-well plate. All samples are tested in triplicate. The plates are sealed with a plastic sheet.

The control viral RNA is prepared to contain 10^6 to 10^7 copies per 3 µl. Serial dilutions of control RNA are prepared using RNAse-free water by adding 5 µl of the control to 45 µL of water and repeating this for 7 dilutions. This gives a standard curve with a range of 1 to 10^7 copies/reaction. The sub-genomic-N uses a known plasmid for its curve. Duplicate samples of each dilution are prepared as described above. If the copy number exceeds the upper detection limit, the sample is diluted as needed. For amplification, the plate is placed in an Applied Biosystems 7500 Sequence detector and amplified using the following program: 48°C for 30 minutes, 95°C for 10 minutes followed by 40 cycles of 95 °C for 15 seconds, and 1 minute at 55°C. A printout of the results is maintained in the laboratory notebook. The number of copies of RNA per ml is calculated by extrapolation from the standard curve and multiplying by the reciprocal of 0.2 ml extraction volume. This gives a practical viral load range of 50 to 5 x 10^8 RNA copies per gram of lung tissue.
sg-N-F: 5'-CGATCTCTTGTAGATCTGTTCTC-3'
sg-N-R: 5'-GGTGAACCAAGACGCAGTAT-3'
Sg-N-P: 5'-6-FAM/TAACCAGAA/ZEN/TGGAGAACGCAGTGGG/3IABkFQ/

Histopathology
Histopathological evaluation of lung sections was performed by Board-certified pathologists at Experimental Pathology Laboratories, Inc. (Sterling, VA) for SARS-CoV-2-related findings. At necropsy, organs were collected and placed in 10% neutral buffered formalin for histopathologic analysis. Tissues were processed through to paraffin blocks, sectioned once at ~5 µm thickness, and stained with hematoxylin/eosin.

References:

1. M. Xu, M. Pradhan, K. Gorshkov, J. D. Petersen, M. Shen, H. Guo, W. Zhu, C. Klumpp-Thomas, S. Michael, M. Itkin, Z. Itkin, M. R. Straus, J. Zimmerberg, W. Zheng, G. R. Whittaker, C. Z. Chen, A high throughput screening assay for inhibitors of SARS-CoV-2 pseudotyped particle entry. *SLAS Discov* **27**, 86-94 (2022).
2. E. Pradenas, B. Trinite, V. Urrea, S. Marfil, F. Tarres-Freixas, R. Ortiz, C. Rovirosa, J. Rodon, J. Vergara-Alert, J. Segales, V. Guallar, A. Valencia, N. Izquierdo-Useros, M. Noguera-Julian, J. Carrillo, R. Paredes, L. Mateu, A. Chamorro, R. Toledo, M. Massanella, B. Clotet, J. Blanco, Clinical course impacts early kinetics,magnitude, and amplitude of SARS-CoV-2 neutralizing antibodies beyond 1 year after infection. *Cell Rep Med* **3**, 100523 (2022).
3. F. R. R. Moreira, M. D'Arc, D. Mariani, A. L. Herlinger, F. B. Schiffler, A. D. Rossi, I. C. Leitao, T. D. S. Miranda, M. A. C. Cosentino, M. C. P. Torres, R. da Costa, C. C. A. Goncalves, D. S. Faffe, R. M. Galliez, O. Junior, R. S. Aguiar, A. F. A. Dos Santos, C. M. Voloch, T. Castineiras, A. Tanuri, Epidemiological dynamics of SARS-CoV-2 VOC Gamma in Rio de Janeiro, Brazil. *Virus Evol* **7**, veab087 (2021).
4. J. Hadfield, C. Megill, S. M. Bell, J. Huddleston, B. Potter, C. Callender, P. Sagulenko, T. Bedford, R. A. Neher, Nextstrain: real-time tracking of pathogen evolution. *Bioinformatics* **34**, 4121-4123 (2018).
5. A. O'Toole, E. Scher, A. Underwood, B. Jackson, V. Hill, J. T. McCrone, R. Colquhoun, C. Ruis, K. Abu-Dahab, B. Taylor, C. Yeats, L. du Plessis, D. Maloney, N. Medd, S. W. Attwood, D. M. Aanensen, E. C. Holmes, O. G. Pybus, A. Rambaut, Assignment of epidemiological lineages in an emerging pandemic using the pangolin tool. *Virus Evol* **7**, veab064 (2021).
6. K. Katoh, D. M. Standley, MAFFT multiple sequence alignment software version 7: improvements in performance and usability. *Mol Biol Evol* **30**, 772-780 (2013).
7. B. Q. Minh, H. A. Schmidt, O. Chernomor, D. Schrempf, M. D. Woodhams, A. von Haeseler, R. Lanfear, IQ-TREE 2: New Models and Efficient Methods for Phylogenetic Inference in the Genomic Era. *Mol Biol Evol* **37**, 1530-1534 (2020).
8. Z. Yang, Maximum likelihood phylogenetic estimation from DNA sequences with variable rates over sites: approximate methods. *J Mol Evol* **39**, 306-314 (1994).
9. Rodon, J., Muñoz-Basagoiti, J., Perez-Zsolt, D., Noguera-Julian, M., Paredes, R., Mateu, L., Quiñones, C., Perez, C., Erkizia, I., Blanco, I., Valencia, A., Guallar, V., Carrillo, J., Blanco, J., Segalés, J., Clotet, B., Vergara-Alert, J., Izquierdo-Useros, N. Identification of Plitidepsin as Potent Inhibitor of SARS-CoV-2-Induced Cytopathic Effect After a Drug Repurposing Screen. *Front Pharmacol* **12**, 646676, (2021).
10. M. R. Boyd, K. R. Gustafson, J. B. McMahon, R. H. Shoemaker, B. R. O'Keefe, T. Mori, R. J. Gulakowski, L. Wu, M. I. Rivera, C. M. Laurencot, M. J. Currens, J. H. Cardellina, 2nd, R. W. Buckheit, Jr., P. L. Nara, L. K. Pannell, R. C. Sowder, 2nd, L. E. Henderson, Discovery of cyanovirin-N, a novel human immunodeficiency virus-inactivating protein that binds viral surface envelope glycoprotein gp120: potential applications to microbicide development. *Antimicrob Agents Chemother* **41**, 1521-1530 (1997).
11. T. Mori, L.G. Barrientos, Z. Han, A.M. Gronenborn, J.A. Turpin, M.R. Boyd, Functional homologs of cyanovirin-N amenable to mass production in prokaryotic and eukaryotic hosts. *Protein Expr Purif.* **26**, 42-49 (2002).
12. B.G. Pierce, K. Wiehe, H. Hwang, B.H. Kim, T. Vreven, Z. Weng, **ZDOCK Server:** Interactive Docking Prediction of Protein-Protein Complexes and Symmetric Multimers. *Bioinformatics* **30**, 1771-1773 (2014) .

2. Supplementary Figure 1. Initial data showing inhibition of SARS-CoV-2 (Wuhan) pseudovirus infection by cyanovirin-N (CV-N). The effect of the protein CV-N on SARS-CoV-2 pseudovirus infection of ACE2$^+$ HEK293 cells measured by luciferase visualization (closed squares, LUC) and its cytotoxic effect on the same ACE2$^+$ HEK293 cells as measured by the reduction in metabolism of the XTT (2,3-Bis-(2-Methoxy-4-Nitro-5-Sulfophenyl)-2*H*-Tetrazolium-5-Carboxanilide) dye (closed circles, XTT). Data shown is the mean of three experiments.

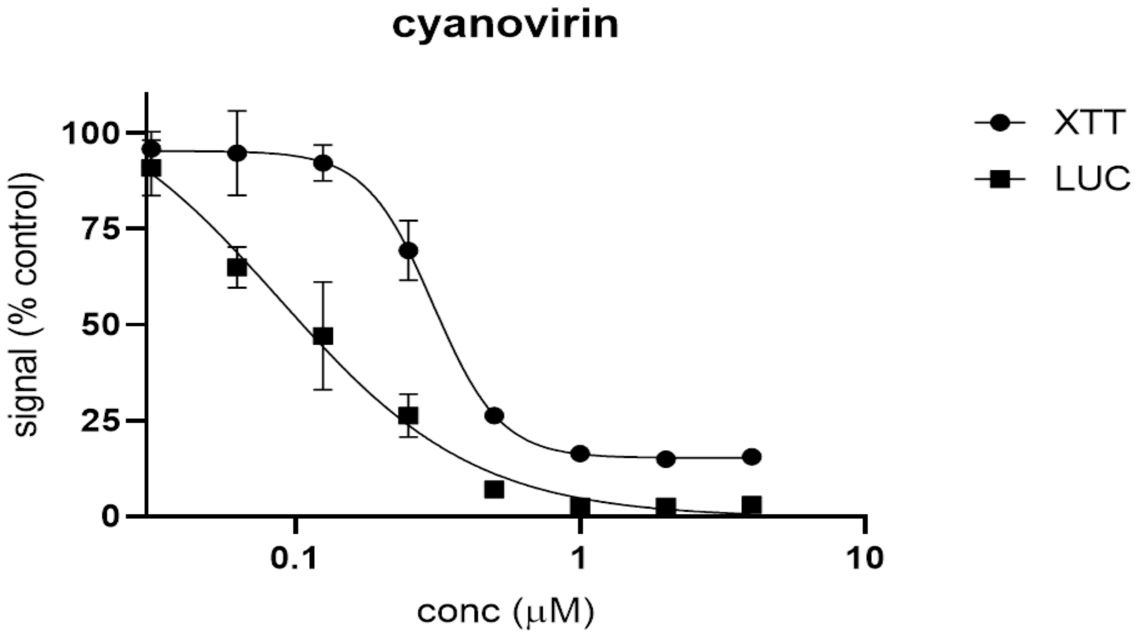

3. Supplementary Table 1. Information on tested SARS-CoV-2 strains

SARS-CoV-2 isolate name	Isolate code [a]	WHO variant [b]	Pango	Aminoacid Substitutions [c]	Amninoacid Deletions [c]	Amninoacid Insertions [c]	Seq. Identifier [d]	Acknowledgements / Reference
**HIAE-02: SARS-CoV-2/SP02/human/2020/BRA**	SP1	-	B	ORF1a:L3606F,ORF3a:G251V	-	-	MT126808.1 *	**Dr. Edison L. Durigon (Laboratory of Clinical and Molecular Virology, Institute of Biomedical Sciences, University of São Paulo)**
**hCoV-19/Brazil/RJ-LVM161-2/2020**	RJ1	-	A.2	N:S197L,ORF1a:F3071Y,ORF3a:G196V,ORF8:L84S,S:I197V	-	-	EPI_ISL_528539	**Dr. Luciana Costa and Dr. Amilcar Tanuri (Laboratory of Molecular Virology, Institute of Biology, UFRJ)**
**hCoV-19/Brazil/RJ-MD63-1a/2020**	RJ2	-	B.1.1.33	N:R203K,N:G204R,N:I292T,ORF1b:P314L,ORF6:I33T,S:D614G	-	-	EPI_ISL_528637	**Dr. Luciana Costa and Dr. Amilcar Tanuri (Laboratory of Molecular Virology, Institute of Biology, UFRJ)**
**hCoV-19/Brazil/RJ-UFRJ-4117/2020**	4117	-	B.1.1.33	N:R203K,N:G204R,N:I292T,ORF1a:S142*,ORF1a:E797K,ORF1b:P314L,ORF6:I33T,S:D614G	-	-	EPI_ISL_12694869	**Dr. Luciana Costa and Dr. Amilcar Tanuri (Laboratory of Molecular Virology, Institute of Biology, UFRJ) - Leitão et al. 2021**
**hCoV-19/Brazil/RJ-UFRJ-6439sw/2020**	6439	-	B.1.1.33	N:R203K,N:G204R,N:I292T,ORF1a:G392C,ORF1a:S1666G,ORF1b:P314L,ORF6:I33T,S:D614G	-	-	EPI_ISL_2134584	**Dr. Luiza Higa and Dr. Amilcar Tanuri (Laboratory of Molecular Virology, Institute of Biology, UFRJ) - Herlinger et al. 2021**
**hCoV-19/Brazil/RJ-00567/2020**	23814	Zeta	P.2	N:A119S,N:R203K,N:G204R,N:M234I,ORF1a:L3468V,ORF1a:L3930F,ORF1b:P314L,S:E484K,S:D614G,S:V1176F	-	-	EPI_ISL_717951	**Dr. Luciana Costa and Dr. Amilcar Tanuri (Laboratory of Molecular Virology, Institute of Biology, UFRJ) - Voloch et al. 2021**
**hCoV-19/Brazil/AM-L70-71-CD1739/2020**	P1-USP	Gamma	P.1	N:P80R,N:R203K,N:G204R,ORF1a:S1188L,ORF1a:K1795Q,ORF1a:S2553F,ORF1a:G3676S,ORF1a:F3677L,ORF1b:P314L,ORF1b:E1264D,ORF3a:S253P,ORF8:E92K,ORF9b:Q77E,S:L18F,S:T20N,S:P26S,S:D138Y,S:R190S,S:E484K,S:N501Y,S:D614G,S:H655Y,S:T1027I,S:V1176F	-	-	EPI_ISL_1060902	**Dr. Lucy S. Villas-Boas and Prof . Maria Cassia Mendes-Correa (Laboratory of Medical Investigation in Virology from University of Sao Paulo) - Faria et al. (2021)**
**hCoV-19/Brazil/RJ-UFRJ-49947/2021**	49947	Delta	AY.99.2	M:I82T,N:D63G,N:R203M,N:G215C,N:D377Y,ORF1a:I1091V,ORF1a:V1187I,ORF1a:A1306S,ORF1a:P2046L,ORF1a:P2287S,ORF1a:T2791I,ORF1a:V2930L,ORF1a:T3255I,ORF1a:S3344A,ORF1a:T3646A,ORF1a:T4087I,ORF1b:P314L,ORF1b:G662S,ORF1b:P1000L,ORF1b:A1918V,ORF3a:S26L,ORF6:M58T,ORF7a:V82A,ORF7a:T120I,ORF7b:T40I,ORF8:F120L,ORF9b:T60A,S:T19R,S:L452R,S:T478K,S:D614G,S:P681R,S:D950N	S:E156-,S:F157-	-	EPI_ISL_12694870	**Dr. Luciana Costa and Dr. Amilcar Tanuri (Laboratory of Molecular Virology, Institute of Biology, UFRJ)**
**hCoV-19/Brazil/SP-HIAE-ID1025/2021**	OmiSP	Omicron	BA.1	E:T9I,M:D3G,M:Q19E,M:A63T,N:P13L,N:R203K,N:G204R,ORF1a:K856R,ORF1a:L2084I,ORF1a:A2710T,ORF1a:T3255I,ORF1a:P3395H,ORF1a:I3758V,ORF1b:P314L,ORF1b:I1566V,ORF9b:P10S,S:A67V,S:T95I,S:Y145D,S:L212I,S:G339D,S:S371L,S:S373P,S:S375F,S:N440K,S:G446S,S:S477N,S:T478K,S:E484A,S:Q493R,S:G496S,S:Q498R,S:N501Y,S:Y505H,S:T547K,S:D614G,S:H655Y,S:N679K,S:P681H,S:N764K,S:D796Y,S:N856K,S:Q954H,S:N969K,S:L981F	N:E31-,N:R32-,N:S33-,ORF1a:S2083-,ORF1a:L3674-,ORF1a:S3675-,ORF1a:G3676-,ORF9b:E27-,ORF9b:N28-,ORF9b:A29-,S:H69-,S:V70-,S:G142-,S:V143-,S:Y144-,S:N211-	-	EPI_ISL_7699344	**Dr.Edison Luiz Durigon (USP), Prof. Ester Sabino (IMT-SP), Fernando Spilke (FEEVALE-SC) and João Renato Rebello Pinho (HIAE)**

4. Supplementary Figure 2. Cyanovirin-N (CV-N) shows activity against various replicating SARS-CoV-2 strains by plaque reduction assay. (A) Plaque reduction neutralization curves of various SARS-CoV-2 strains previously or currently circulating performed in Vero E6 cells. Different viral isolates are distinctly colored (mean of at least four independent experiments).

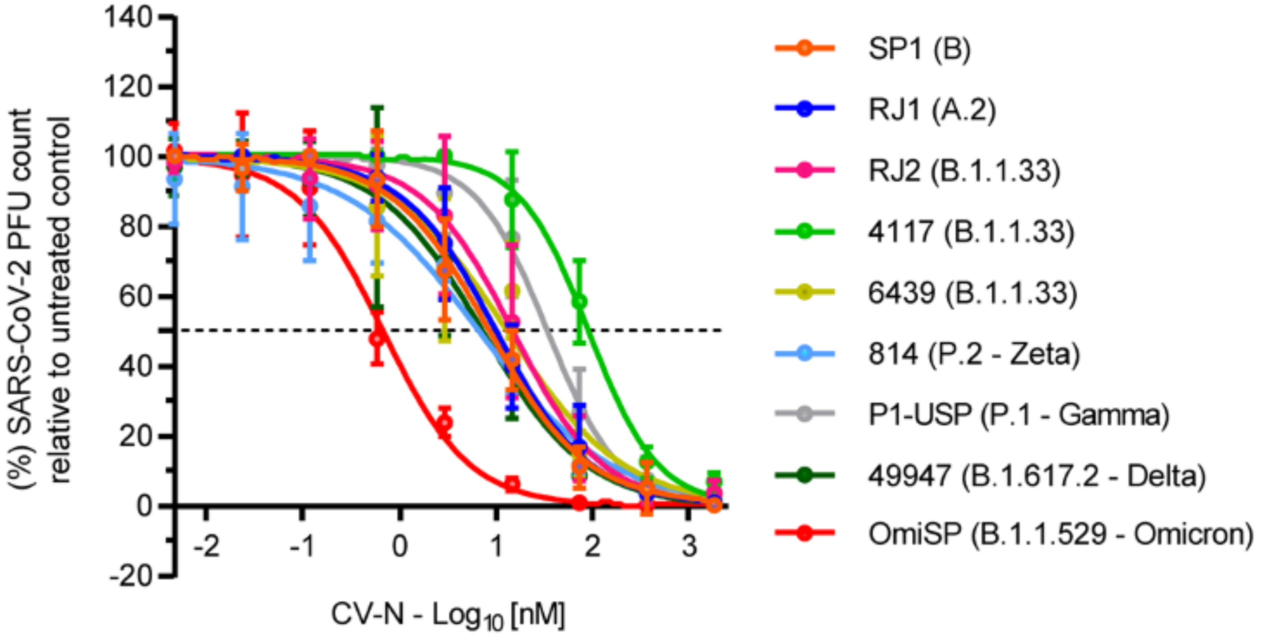

5. Supplementary Table 2. ITC results of CV-N binding to baculovirus and HEK293 cell produced SARS-CoV-2 Spike

	Enthalpy, DH (kcal/mol)	Entropy, TDS (kcal/mol)	Free Energy, DG (kcal/mol)	Affinity, Kd (nM)	Stoichiometry (antiviral protein:CoV2 Spike)
**Baculovirus-produced SARS-CoV-2 Spike**	– 27.0 ± 0.28	– 16.13 ± 0.28	– 10.90 ± 0.06	18.9 ± 1.2	2.40 ± 0.8
**HEK293 cell-produced SARS-CoV-2 Spike**	– 15.70 ± 0.21	– 5.20 ± 0.21	– 10.50 ± 0.04	28.1 ± 1.9	2.04 ± 0.23

6. Supplementary Table 3. Syrian golden hamster experimental design.

Group	N (M/F)	Treatment (IN)	Tx Days	SD 0 Dose (in 100uL)	SD 1-4 Doses (in 100uL)	Challenge (SD0)	Tissue Collection
1	6 (3M/3F)	MEM Mock Control	Immediately Pre-Challenge (~5min), BID on SD 1-4	MEM only	MEM only	N/A	SD 7: Collect lungs and nasal turbinates<br><br>NOTE: Weigh whole lungs prior to sectioning and fixation
2	6 (3M/3F)	CV-N		1 mg/kg	1 mg/kg	SARS-Cov-2 Intranasal	
3	6 (3M/3F)	CV-N		0.2 mg/kg	0.2 mg/kg		
4	6 (3M/3F)	CV-N		0.04 mg/kg	0.04 mg/kg		
5	6 (3M/3F)	MEM Challenged Control		MEM only	MEM only		

7. Supplementary Figure 3. Clinical and virologic evaluation of the pilot experiment for CV-N dosing on Syrian golden hamster following SARS-CoV-2 i.n. challenge. Change in body weight of hamsters was measured daily for unchallenged control (MEM mock control), SARS-CoV-2 (WH-1) challenged control (MEM challenged control), and hamsters treated with either 0.04, 0.2 or 1.0 mg/kg cyanovirin- N (CV-N) for entire study group **(A)**, Females only **(B)**, or males only **(C)**. Viral Challenged control animals displayed mild to marked bronchiolo-alveolar hyperplasia consolidating the lung with mild mixed cell inflammation and syncytial cells **(D)**. Lung tissue from hamsters treated with 0.2 mg/kg CV-**N** showed mild bronchiolo-alveolar hyperplasia scattered throughout the lung with open airways still visible compared to viral challenged control animals **(E)**.

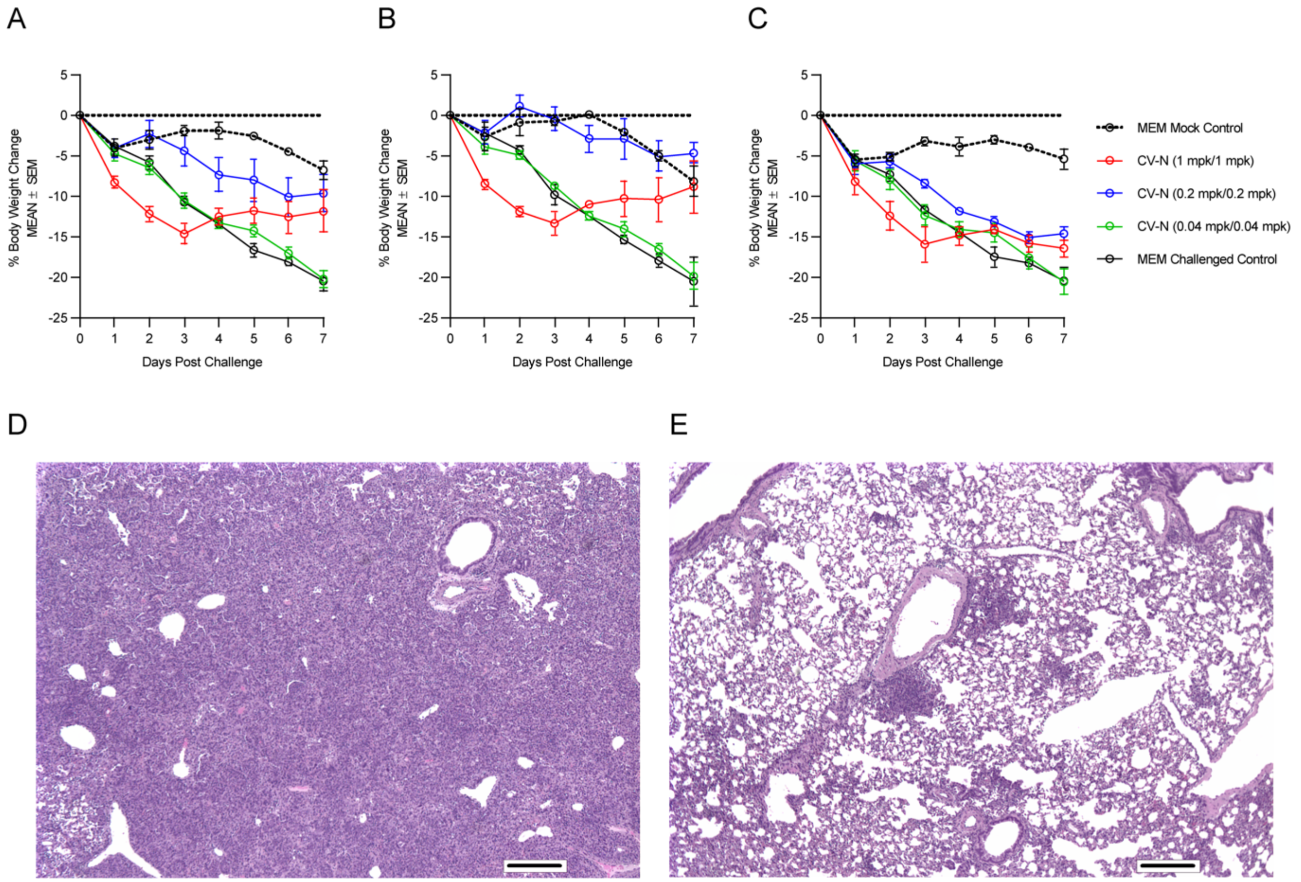

8. Supplementary Table 4. Syrian golden hamster CV-N "*one-shot*" treatment experimental design.

Group	N	SD 0 Treatment (i.n.) (immediately pre challenge)	Challenge (i.n.)	Tissue Collection	
1	12 (6F/6M)	MEM sham treatment	SD 0	SD 4 N=6/group Collect Lungs for RT-PCR	SD 7 N=6/group Collect Lungs for RT-PCR and histopathology
2	12 (6F/6M)	2 mg/kg CV-N	SD 0		

9. Supplementary Figure 4. A single cyanovirin-N (CV-N) intranasal administration protects both female and male Syrian golden hamsters from weight loss following SARS-CoV-2 challenge. Both female **(A)** and male **(B)** hamsters treated with a single dose of 2.0 mg/kg CV-N all showed improvement in body weights when compared to viral challenges control animals.

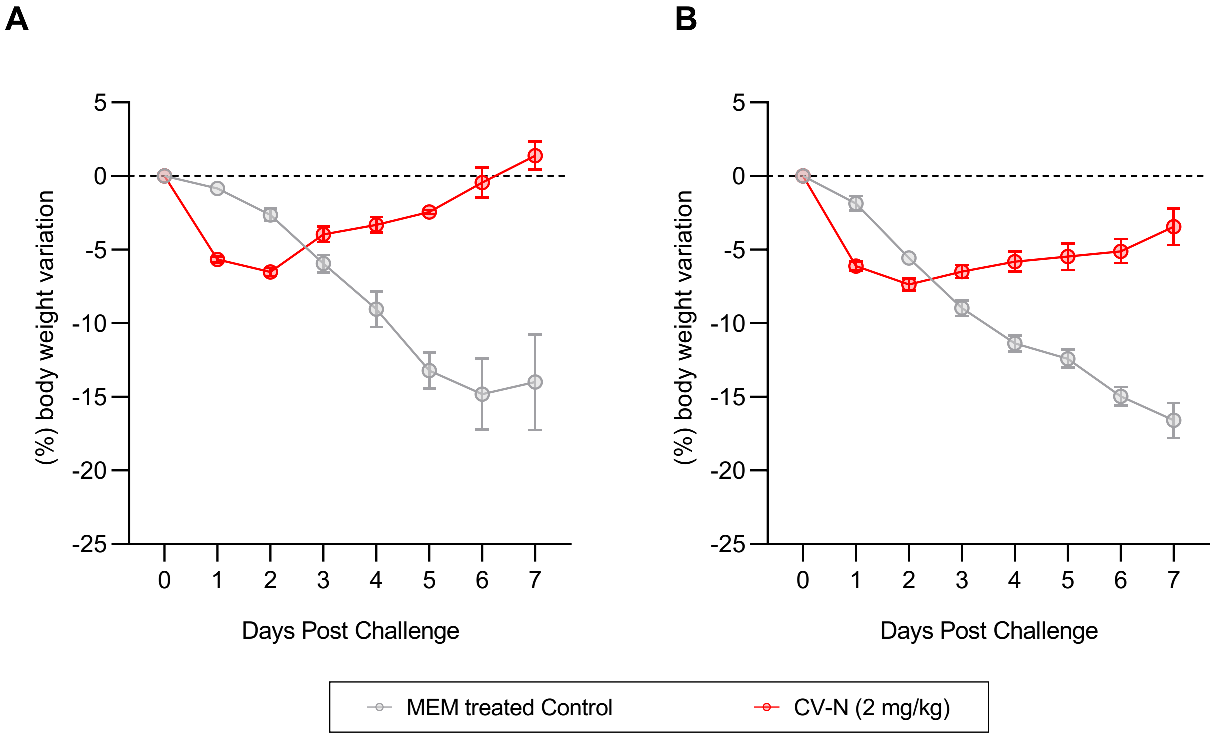

Supplement: Supplementary file 1 — Appendix 01 (PDF) [file pnas.2214561120.sapp.pdf]
